# Research Progress on the Biological Activities of Metal Complexes Bearing Polycyclic Aromatic Hydrazones

**DOI:** 10.3390/molecules27238393

**Published:** 2022-12-01

**Authors:** Ruixue Liu, Jingbo Cui, Tongyan Ding, Yancheng Liu, Hong Liang

**Affiliations:** 1 State Key Laboratory for the Chemistry and Molecular Engineering of Medicinal Resources, School of Chemistry & Pharmaceutical Sciences, Guangxi Normal University, Guilin 541004, China; 2 Department of Chemistry, Southern University of Science and Technology, Shenzhen 518055, China

**Keywords:** hydrazone, polycyclic aromatic hydrazone, metal complex, biological activity

## Abstract

Due to the abundant and promising biological activities of aromatic hydrazones, it is of great significance to study the biological activities of their metal complexes for the research and development of metal-based drugs. In this review, we focus on the metal complexes of polycyclic aromatic hydrazones, which still do not receive much attention, and summarize the studies related to their biological activities. Although the large number of metal complexes in phenylhydrazone prevent them all from being summarized, the significant value of polycyclic aromatic hydrocarbons themselves (such as naphthalene and anthracene) as pharmacophores are also considered. Therefore, the bioactivities of the metal complexes of naphthylhydrazone and anthrahydrazone are focused on, and the recent research progress on the metal complexes of anthrahydrazone by the authors is also included. In terms of biological activities, these complexes mainly show antibacterial and anticancer activities, along with less bioactivities. The present review demonstrates that the structural design and bioactivities of these complexes are fundamental, which also indicates a certain structure—activity relationship (SAR) in some substructural areas. However, a systematic and comprehensive conclusion of the SAR is still not available, which suggests that more attention should be paid to the bioactivities of the metal complexes of polycyclic aromatic hydrazones since their potential in structural design and biological activity remains to be explored. We hope that this review will attract more researchers to devote their interest and energy into this promising area.

## 1. Introduction

Various organic compounds can exert rich and diverse biological and pharmacological activity by virtue of their different pharmacophores and functional groups, such as quinoline, anthraquinone, and porphyrin as large groups and/or hydroxyl, carboxyl, and imine as small groups. They play pivotal roles through weak intermolecular forces such as hydrogen bonds, π-π stacking, and even stronger covalent bonds, while they act on large molecular targets with different shapes and functions [1]. Among these functional groups, aromatics (which include the most basic benzene rings, polycyclic aromatics, and even aromatic heterocyclics) are some of the most important target pharmacophores in molecular biology research and drug design, as their significant delocalized conjugated structures play a pivotal role in exerting pharmacological activity [2]. Polycyclic aromatic hydrocarbons further extend this super-conjugated structure, which makes them more biologically active [3,4]. Generally, polycyclic aromatic hydrazones, depending on their planar conjugated system, are regarded as being able to intercalate/be inserted between DNA base pairs, so they are often used as DNA intercalators/inserters to exert their anticancer activity [5]. In addition, the metal complexes of polycyclic aromatics can also be used as DNA probes for medical applications. For example, J. K. Barton et al. previously carried out the probe identification and reaction of the corresponding transition metal complexes applied to double-helix DNA, focusing on the role of DNA charge-transfer chemistry in DNA repair [6,7,8]. They explored the different properties of DNA and RNA by pairing different central metal ions such as Ru and Rh with various commercially available or custom-made ligands to form metal complexes of different structures as probes and using the photochemical properties of these metal ions to induce nucleic acid cleavage. Specifically, the binding pattern and specificity of the complexes could be used to explore the groove width and depth of the DNA as well as the possible existence of unusual structures and the tertiary folding patterns of nucleic acids.

Meanwhile, as the number of rings increases, the corresponding toxicity and side effects may also increase. Thus, the derivatives of anthracene or phenanthrene have been found to be more toxic than those of naphthalene. Therefore, how to balance the activity and toxicity while increasing the number of rings is also a special consideration and focus in designing novel compounds. Another interesting group is hydrazone, “-C=N-NH-”, which can also act as an extended but more stable imine bond, “C=N”. Additionally, with the addition of one more N atom, it exhibited more abundant biological activity and coordination potential [9,10,11]. The aromatic hydrazone (arylhydrazone), which contains the pharmacophores of both the aromatic ring and hydrazone, can produce more abundant and complex pharmacological activities [11]. Phenylhydrazone is the simplest arylhydrazone and can be obtained via the condensation of benzaldehyde with a hydrazine derivative. The condensation products of hydrazine and naphthyl aldehyde can obtain 1-naphthylhydrazone or 2-naphthylhydrazone because of the different positions of the aldehyde group on naphthalene, although 1-naphthylhydrazone is the predominant one. Up until now, a large number of metal complexes of (phenyl/naphthyl)thiosemicarbazone have been reported as potential antimicrobial agents or anticancer candidates. As early as the 1950s, thiosemicarbazone was found to inhibit the activity of RNA reductase and to thus exhibit significant anticancer activity [12,13]. In addition, thiosemicarbazone itself is a type of satisfying chelate ligand, so its metal complexes with potential pharmacological activity have been fully explored and studied in recent years [14,15,16]. For example, Valentina Gandin et al. reported a series of copper(II) complexes with salicylaldethiosemicarbazone as ligands and found that they showed significant inhibitory activity against the colon cancer cell line LoVo and its oxaliplatin-resistant strains, with IC_50_ values ranging from 0.004 to 0.036 μM, reaching the nmol level [15]. They also showed a good inhibitory effect on the 3D cell spheres of colon cancer cells (HCT-15) and pancreatic cancer cells (PSN1). J. Y. Niu et al. reported a series of Mn/Co/Zn complexes of thiosemicarbazone, which showed significant anticancer activity against the K562 leucocythemia cancer cell line [16]. Aromatic hydrazones with more than three conjugated rings (e.g., anthracene, phenanthrene, pyrene, etc.) are relatively rare, which is due to the fact that their corresponding aromatic aldehydes are much less studied. In particular, bisantrene, a symmetric derivative of 9, 10-anthracene dihydrazone, was successfully developed as a novel anticancer drug and started to be applied in clinical chemotherapy in the 1990s. It is also acted as a derivative of anthracycline anticancer drugs [17].

On the other hand, studying the biological activity of metal complexes is attractive to many researchers, especially to inorganic chemists. Metal complexes can exert both the activity of the organic ligand and the metal center through coordination and can even achieve a positive synergistic effect to obtain better biological activity [18]. Admittedly, the ligands of many metal complexes might not show any potential biological activity and may mainly act as carriers by coordinating with the central metals to carry or wrap them to the target cell or tissue region to exert their activities [19]. Additionally, it should be also admitted that the formation of highly active metal complexes in polycyclic aromatics may also bring about varying degrees of toxicity or side effects [20]. Nevertheless, the activity level of the metal complexes is closely related to the role and biochemical function of the central metal ions themselves. For example, copper is an essential trace element in the life process and plays an important role in maintaining the redox cycle capacity of living systems [21,22]. Y. Gou et al. recently reported three fluorescent dithiocarbazate—copper complexes that showed significantly higher cytotoxicity to several pancreatic cancer cells than ligands and cisplatin [23]. Confocal fluorescence imaging showed that one of the complexes, complex **3**, which was primarily targeted at mitochondria, was able to kill pancreatic cancer cells by triggering multiple mechanisms. It was reported to be the first copper(II) complex to trigger the ferroptosis pathway in cancer cells. In addition, iridium(III) complexes with different functions have been reported to trigger the ferroptosis pathway by targeting mitochondria or lysosomes [24,25] or to trigger light-induced ferroptosis in hypoxic tumor cells through photodynamic pathways, leading to cancer cell death [26]. Obviously, bifunctional complexes based on “active metal + active ligand” should be emphasized as a hot research direction and as the foundation of metal complex research, as they show more design space and selectivity [27].

As mentioned above, aromatic hydrazone compounds have exhibited significant and extensive bioactivities. Thus, more and more attention has been paid to the research on metal complexes with an aromatic hydrazone as the active ligand in recent years, with the abundant research reports on the metal complexes of phenylhydrazone being due to the simple availability and large number of phenylhydrazone derivatives [28,29,30]. In comparison, polycyclic aromatic hydrazones were found to be able to obtain more significant aromatic planar properties by further extending the hyperconjugated structure of the benzene ring. Therefore, relatively less attention has been paid to the metal complexes of polycyclic aromatic hydrazones, especially according to the reports on their biological activities. It is of great significance to further study the metal complexes of polycyclic aromatic hydrazones by considering their potential medicinal prospects. However, the related commentary on their research progress and orientation remains undeveloped. Thus, based on the continuous work and efforts by the authors on the metal complexes of anthrahydrazone as well as on their anticancer activity in recent years, the recent progress surrounding the metal complexes of polycyclic aromatic hydrazones (such as naphthylhydrazone, phenanthrylhydrazone, and anthrachydrazone) and their biological activities was reviewed for the first time [31,32,33]. We hope this work presents a primary overview of this research field, which is still less focused on and less systematic, to relevant researchers. Through the existing research work, some possible rules of the structure–activity relationship could be summarized and more promising exploration directions were found, providing a reference and basis for better and higher-level research exploring the bioactive metal complexes of polycyclic aromatic hydrazones.

## 2. The Antibacterial Activity Study

### 2.1. Antibacterial Metal Complexes of Naphthylhydrazone

Hydrazones have been widely used in medicine, agricultural chemicals, functional materials, etc., due to their potential pharmacological activities, including anticancer [34,35,36], antibacterial [37,38], anti-inflammatory [39,40], and antiviral [41,42] effects, among others. Naphthalene is a double ring formed by two benzene rings in parallel and is the simplest polycyclic aromatic hydrocarbon. It has long been attempted to introduce a naphthalene ring into the structure of hydrazone to form a new hydrazone with distinct structural characteristics and potentially rich pharmacological activities, among which antibacterial and anticancer activities are the most extensive.

#### 2.1.1. The First Transition Metal Complexes of Naphthylhydrazone with Antibacterial Activities

**Figure 1:**
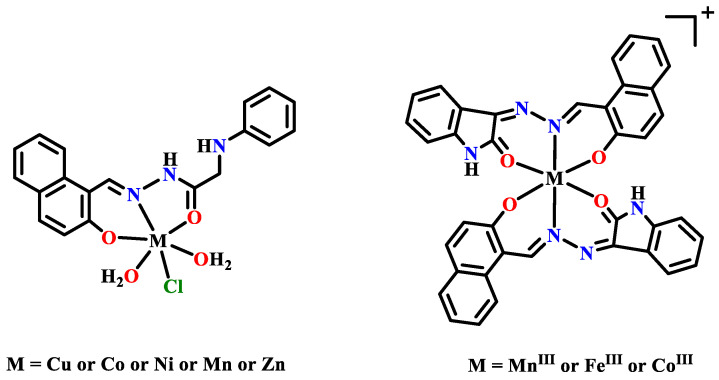
Some representative first transition metal complexes of naphthylhydrazone with *N*-phenylamino derivatives as the side chain.

Kalagouda B. Gudasi et al. [43] first synthesized and characterized the novel naphthylhydrazone ligand with *N*-phenylglycine as its side chain as well as its copper(II), nickel(II), cobalt(II), manganese(II), and zinc(II) complexes in 2006 (Figure 1). All of these complexes showed an octahedral geometry of the metal center and similar coordination characteristics, as suggested by spectroscopic studies. The spectral results speculated that the naphthylhydrazone ligand tridentate coordinated with the metal ions via deprotonated naphtholate-O, azomethine-N, and carbonyl-O. The antibacterial and antifungal activities of the complexes showed that they all exhibited stronger and broader-spectrum biological antibacterial activity than the ligand or the metal salts alone. Then, Balakrishnan Murukan et al. [44] reported the new naphthalyl-dihydrazone in 2007, which is formed by the condensation of isatin monohydrazone with 2-hydroxy-1-naphthaldehyde to afford its new manganese(III), iron(III), and cobalt(III) complexes (Figure 1). The spectral analysis indicated that the naphthalene-dihydrazone ligand coordinated with the transition metal ions by deprotonated naphtholate-O, azomethine-N, and carbonyl-O atoms. The coordination center of these complexes was suggested to form an octahedral geometry according to infrared spectroscopy (IR) and nuclear magnetic resonance (NMR) spectral analysis. The in vitro antibacterial activities of the compounds against several bacterial strains were determined. The results showed that the antibacterial activity of the ligand was weaker than the complexes. The antibacterial activity of the complex was similar to that of the complexes with Schiff’s base, hydrazone, or thiosemicarbazone as the corresponding ligands, as reported in previous literature. It was speculated that the chelation between the ligand and metal ions could greatly reduce the polarity of the metal ions and increase the lipophilicity of the whole complex to facilitate its interaction with lipid substances (such as the cell membrane and macromolecular hydrophobic regions).

In 2008, A. P. Gulya et al. [45] reported the synthesis and antibacterial activity of a series of copper(II) complexes with naphthalidenethiosemicarbazone and sulfanilamide as co-ligands. The influences of Cu(II) and the phenyl group on the skeleton of thiosemicarbazone on their antibacterial activity towards 10 different G+/G− bacteria strains were discussed, in which the coccal test strains showed the highest sensitivity towards the Cu(II) complexes bearing norsulfazole, ethazole, or sulfadimezine groups as co-ligands. Additionally, the intraspherical ligands affected both their bacteriostatic and bactericidal abilities directly. Under the modulated side chain of thiosemicarbazone or the type of co-ligands, the minimum inhibitory concentration and microbial biomass carbon (MIC and MBC) values increase (corresponding to decreased antibacterial activity), such as when the phenyl group of naphthylhydrazone is removed, or the streptocide or sulfacyl group takes the place of norsulfazole, ethazole, or sulfadimezine. These results suggest the potential for seeking antibacterial drugs from the bio-metal complexes of naphthyl-thiosemicarbazone.

**Figure 2:**
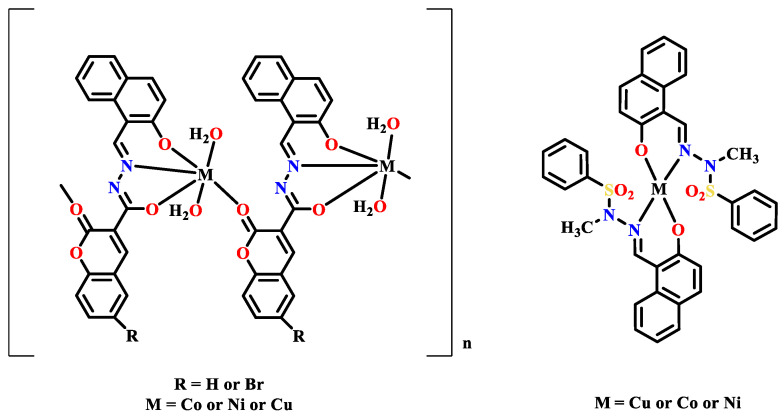
The Cu(II)/Co(II)/Ni(II) complexes of naphthylhydrazone with *N*-methyl benzene sulfonamide as side chain.

Sangamesh A. Patil [46] further studied a series of Co(II)/Ni(II)/Cu(II) complexes bearing novel naphthalylhydrazones with coumarin-type side chains in 2011 (Figure 2). It was found that all of these metal complexes showed a six-coordinated octahedral geometry by the tridentated hydrazone ligand via phenolic-O, azomethine-N, and amide carbonyl-O atoms; two H_2_O; and lactonyl O from another hydrazone ligand to form a one-dimensional chain polymer. These complexes were soluble in dimethyl formamide (DMF) or dimethyl sulfoxide (DMSO) and melted at higher temperatures. Electrochemical studies of the Cu(II) and Ni(II) complexes showed that they were one-electron-transfer *quasi*-reversible redox pairs. It was also found that the metal complexes had stronger antibacterial activity against some typical bacteria (*E. coli*, *S. aureus*, *B. subtilis*, and *S. typhi*) and fungi (*C. albicans*, *A. Niger*, and *Cladosporium*) than the ligand, indicating that the synergistic action of the ligand and metal ions significantly enhanced the antibacterial activity. Then, H.G. Aslan et al. [47] reported new Co(II), Ni(II), and Cu(II) complexes of 2-hydroxy-1-naphthylhydrazone bearing benzenesulfonyl on the side chain in 2013, as shown in Figure 2. Unfortunately, these complexes showed lower antibacterial activity than the ligand alone against a series of bacteria strains, including both G+- and G−-type, and even fungi, which was not consistent with the trend of increased activity of the complexes reported before. Thus, it should be noted that the antibacterial activity of the metal complex of naphthylhydrazone is not unidirectionally enhanced by the coordination of metal ions with the ligand. The antibacterial activity of the complex might depend on the potential synergistic effect between the ligand and the metal ion, and the specific coordination mode or the corresponding existing species in solution should be also considered.

**Figure 3:**
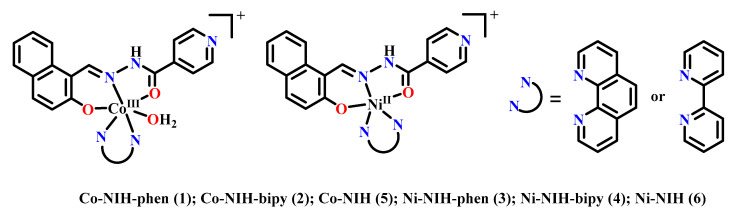
The Co(III)/Ni(II) complexes of NIH (2-hydroxy-1-naphthaldehyde isonicotinoyl hydrazone).

Meanwhile, K.R. Sangeetha Gowda et al. [48] reported six Co(III) and Ni(II) complexes of NIH (2-hydroxy-1-naphthaldehyde isonicotinoyl hydrazone) by introducing the typical *N*,*N*′-bidentate co-ligand (phen or bipy) (Figure 3). The central Co(III)/Ni(II) was six- and five-coordinated by the tridentate 2-hydroxy naphthylhydrazone via the phenolic- and amide carbonyl-O atoms and the azomethine N atom, along with bipyridine and *o*-phenanthroline, respectively. The deoxyribonucleic acid (DNA)-binding properties of these complexes were also studied by spectral analyses and in DNA viscosity experiments. The results indicated that those complexes bearing phen or bipy as the co-ligand showed stronger intercalative DNA-binding abilities towards DNA as well as DNA photo-cleavage potentials. In view of their *K*_b_ values, the two Co(III) complexes of NIH bearing phen (**1**) or bipy (**2**) were 4.6 × 10^4^ and 4.1 × 10^4^ M^−1^, and those for the corresponding two Ni(II) complexes (**3** and **4**) were 4.9 × 10^4^ and 4.2 × 10^4^ M^−1^, which were obviously higher than the Co(III) (**5**) or Ni(II) (**6**) complexes of NIH alone, with *K*_b_ only being 3.6 × 10^4^ M^−1^ and 2.8 × 10^4^ M^−1^, respectively. Accordingly, their antibacterial activities were found to be basically coincident with the respective *K*_b_ values, further implying the key roles of both the metal center and the co-ligand (phen or bipy), which are believed to be responsible for the improved bioactivities [49,50]. Nevertheless, the differences between the metal center Co(III) and Ni(II) were not further reflected in the DNA-binding ability and in vitro antibacterial activity. Although the two co-ligands (phen/bipy) also did not show differences in their antibacterial activity, the complex with phen did exhibit stronger DNA-binding ability, which should be related to its larger planar conjugated area.

Additionally, Omima M.I. Adly et al. [51] synthesized a similar type of 2-hydroxy-1-naphthylhydrazone bearing a 1,2,4-triazin side chain. Since a 3-thioxo group exists on the triazin, this ligand coordinated to the metal center (Cu(II), Co(II), Ni(II), Zn(II), Cd(II), VO(IV), and UO_2_(VI)) in a tridentate mode via phenolic-O, azomethine-N, and thioxo-S. Although the coordination geometry showed diversity, such as planar, tetrahedral, pyramidal, or octahedral diversity, all of the transition metal complexes formed in the mononuclear type, as determined by ultraviolet—visible (UV—Vis), electron spin resonance (ESR), and magnetic analysis. They were also tested to determine their in vitro antibacterial activity towards the typical G+ (*S. aureus* and *B. subtilis*) and G− (*S. typhi* and *E. coli*) strains as well as towards the fungi *C. albicans*. Although most of the complexes showed obviously higher antibacterial activity than the ligand, their activity was still intermediate or low (<2/3 of mean zone diameter of the corresponding positive control), except for the Cd(II) complex (Cd(HL)(NO_3_)), which showed comparable activity to the positive control towards *B. subtilis* and *S. typhi*.

**Figure 4:**
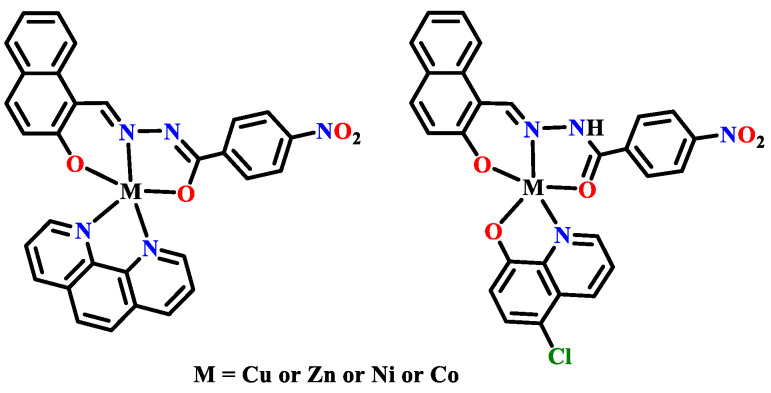
The transition metal complexes of naphthylhydrazone with *p*-nitrobenzamide as a side chain together with *o*-phenanthroline or 5-chloro-8-hydroxyquinoline as the co-ligand.

Eight transition metal complexes (Co(II), Ni(II), Cu(II), and Zn(II)) were also synthesized from a 2-hydroxy-1-naphthylhydrazone ligand bearing a *p*-nitrobenzamide side chain, and *o*-phenanthroline or 5-chloro-8-hydroxyquinoline was introduced as the co-ligand by Ganga K. Rajam et al. [52], the structure of which was analyzed by means of X-ray diffraction (XRD), as indicated in Figure 4. Compared with the ligand, the antibacterial experiments showed that the complexes had enhanced antibacterial activity but were still inferior to the positive control drug, Endofill. The proliferation of *B. subtilis* and *S. aureus* was highly inhibited at the MIC value of 200 μg/mL, while *E. coli* and *P. putida* were not significantly inhibited in the presence of these complexes. Alternatively, the antifungal activity of the complexes was found to be poor, with the MIC values being about as high as 1 mg/mL, and no obvious antifungal activity was observed at lower concentrations.

**Figure 5:**
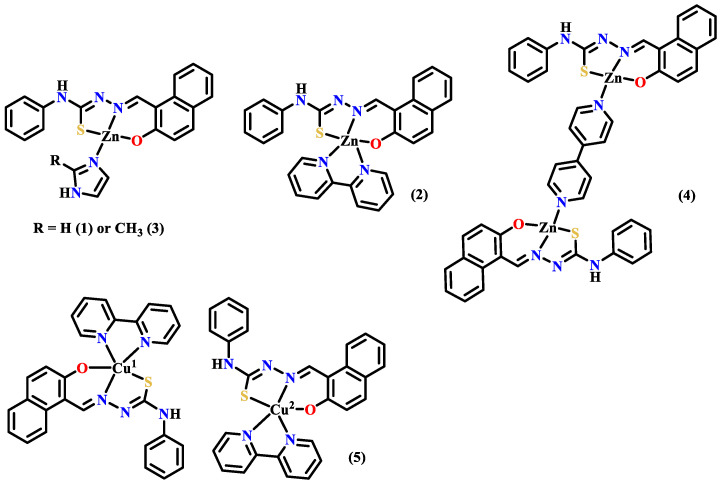
The Cu(II)/Zn(II) complexes of naphthylhydrazone with phenylthiourea as side chain together with several *N*-co-ligands.

Sedaghat’s group also reported four Cu(II)/Zn(II) complexes of 2-hydroxy-1-naphthylhydrazone with a phenylthiosemicarbazone side chain by introducing 4,4′-bipy (*N*,*N*′-bidentate), imidazole, or 2-methylimidazole (*N*-monodentate) as the co-ligand [53]. The hydrazone ligand tridentated to the center Cu(II) or Zn(II) via the deprotonated phenolic-O and thiol-S as well as via the azomethine-N atom together with the auxiliary co-ligand N atom to form the coordination sphere of the metal complex in the electric neutral state. As the co-ligand, bipy could help to form a binuclear complex, such as complex **4** in Figure 5. The metal complexes showed significantly higher antibacterial activity than the hydrazone ligand towards the tested *B. subtilis*, *S. aureus*, and *P. aeruginosa* strains. For copper(II), complex **3,** in particular, the inhibition zone against *P. aeruginosa* reached 30 mm, which suggested far more sensitivity to **3** than the ligand as well as to the two positive controls, nalidixic acid and vancomycin.

**Figure 6:**
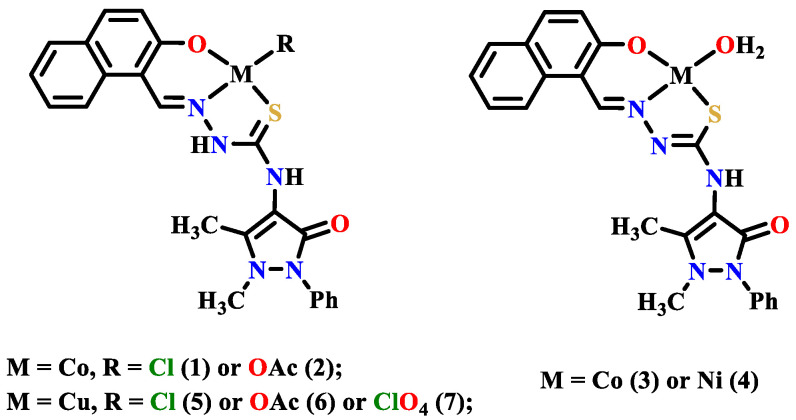
The Cu(II)/Co(II)/Ni(II) complexes of naphthylhydrazone with antipyrine-type side chain.

Recently, Ayman K. El-Sawaf et al. [54] reported a series of mononuclear Co(II)/Ni(II)/Cu(II) complexes of 2-hydroxy-1-naphthylhydrazone with an antipyrine side chain, which was synthesized by condensation on the introduced 4-amino of antipyrine (Figure 6). The hydrazone ligand in this work also coordinated to the metal center via O/N/S atoms, similar to the complexes mentioned above [53], but the S atom of the thione group (C=S) was in the electroneutral state, so the co-ligand R involved in the coordination sphere was belonged to a −1-charged group, such as Cl^−^, OAc^−^, or ClO_4_^−^. Two typical bacteria strains, *S. aureus* and *E. coli*, were chosen to test the antibacterial activities of these compounds based on the inhibition zone. It was found that all seven transition metal complexes showed higher activity than this hydrazone ligand bearing an antipyrine side chain, although they were still not as active as enrofloxacin. Similarly, the copper(II) complex **5** resulted in the biggest inhibition zone, with diameters of 14.8 and 16.1 mm against *S. aureus* and *E. coli*, respectively. The antibacterial activity results also showed that in addition to the differences in the metal centers, the difference in the −1-charged R group also significantly affected the activity of the complexes, which might be related to the dissociation kinetics of the complex in solution.

**Figure 7:**
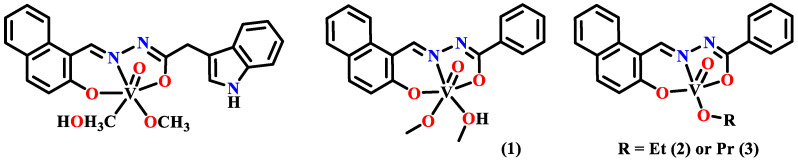
Several vanadium (V) complexes of naphthylhydrazone with indole-3-acetamide group or benzamide as a side chain.

The vanadium complexes of naphthylhydrazone were not explored by W. Li et al. until 2015 [55]. They synthesized the first V(V) complex of 2-hydroxy-1-naphthylhydrazone by introducing the indole-3-acetamide group as the side chain so that an *O*/*N*/*O*-tridentate coordinated the mode of the ligand to the V=O moiety, as indicated in Figure 7. Both the results of the inhibition zone and MIC values clearly indicate that this vanadium complex demonstrated higher inhibition activity during the proliferation of different typical strains, including *E. coli*, *P. aeruginosa*, *B. subtilis*, and *S. aureus*. Towards *P. aeruginosa*, the inhibition zone of the complex was three times larger than that of the ligand, and the MIC value was only one-quarter that of the ligand. However, it was still not as active as the positive control, penicillin G. Then, S. Yousef Ebrahimipour et al. [56] reported three very similar vanadium(V) complexes of 2-hydroxy-1-naphthylhydrazone with a benzamide side chain in 2016 (Figure 7), which were fully characterized on the structures. The coordination mode of these complexes was similar to those reported by Li et al. In this work, they explained the transformation of the ligand from the *keto*-form to the deprotonated *enol*-form under the coordination to V=O along with the deprotonated MeOH/EtOH/n-PrOH, which acted as the co-ligand. The antibacterial activities of the three complexes ([VO(L)(MeOH)(OMe)] (**1**), [VO(L)(OEt)] (**2**) and [VO(L)(OPr)] (**3**)) towards *E coli*, *S. aureus*, and methicillin-resistant *S. aureus* (MRSA) were determined. The results showed that they all had significant antibacterial activities at higher concentrations of 500 and 1000 μg/well. In particular, complex **1** was also highly sensitive to the MRSA-resistant strain, and its corresponding inhibitory zone (30.5 and 28 mm) was higher than that of normal *S. aureus* (26 and 20 mm). Moreover, this antibacterial activity of complex **1** was significantly stronger than that of the two positive controls (cefixime and azithromycin), whose inhibition zones against MRSA were only 12 mm and 15.5 mm, respectively. A molecular docking study showed that the docking energy and binding energy of complex **1** to the key antibacterial target, glCN-6-P synthase, were −8.46 and −5.33 kcal·mol^−1^, respectively, indicating that complex **1** is a good inhibitor of GLCN-6-P synthase.

**Figure 8:**
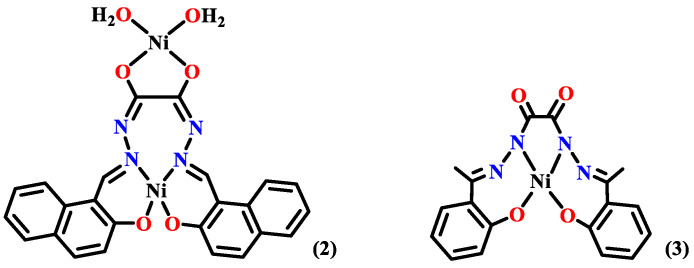
The new binuclear nickel(II) complex bridge coordinated by a special dihydrazone linked by oxamide.

On the other hand, Ayman H. Ahmed et al. [57] synthesized a novel dihydrazone of 2-hydroxy-1-naphthylhydrazone linked by oxalate. Additionally, a new binuclear nickel(II) complex was achieved, which was coordinated by the bridging ligand of dihydrazone linked by oxamide, as shown in Figure 8. Meanwhile, three new nickel(II) complexes of 2-hydroxyphenylhydrazone were also synthesized for comparison, but they were all mononuclear. The antimicrobial results indicated different sensitivities of the five tested bacteria (*B. subtilis*, *S. pneumoniae*, *E. coli*, *S. racemosum*, and *A. fumigatus*) against these nickel(II) complexes. The dihydrazone ligand L2 exhibited the highest activity among the hydrazone ligands. Ni(II) complex **3** showed higher inhibition on *B. subtilis*, *S. pneumoniae*, and *E. coli*, while complex **2** showed a better effect on *S. racemosum* and *A. fumigatus*. However, there was not much difference in the overall antibacterial activity between the Ni(II) complexes and the ligands. It was deduced that these compounds inhibited the microbes by blocking the metal-binding sites of microbial enzymes. They could also interfere with cell respiration, preventing protein synthesis and further limiting microbial proliferation. Since the nickel(II) center was generally not regarded as being the most effective in terms of antimicrobial activity, more transition metal complexes of these ligand types should be encouraged for further exploration.

#### 2.1.2. The Second and Third Transition Metal Complexes of Naphthylhydrazone with Antibacterial Activities

**Figure 9:**
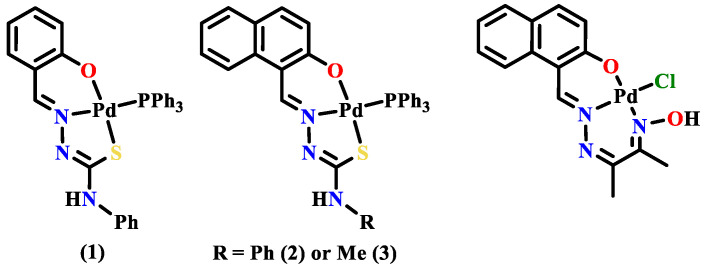
Three palladium(II) complexes of some *N*-substituted aromatic hydrazones.

Early in 2008, R. Prabhakaran et al. [58] synthesized and characterized three palladium(II) complexes of 2-hydroxynaphthylhydrazone (phenylhydrazone was also involved) containing *N*-substituted thiosemicarbazide as a side chain (Figure 9). In each complex, the ligand was also coordinated with the central Pd(II) via the *O*/*N*/*S*-tridentate coordination mode. By comparing the in vitro antibacterial activity of these complexes, it was found that the activity of *N*-phenyl-substituted hydrazone Pd(II) complex **2** was significantly higher than that of *N*-methyl-substituted hydrazone Pd(II) complexes **3** and **1**, so the overall antibacterial activity order was as follows: **2** > **1** > **3**. These results indicated that the different ligand substituents could significantly affect the overall antibacterial activity of the complexes. For complex **2**, the authors also discussed the possible SAR, suggesting that its high activity was related to the co-existence of two electron-withdrawing groups, naphthalene and benzene, and the chelation of the hydrazone ligand also greatly reduced the polarity of the central metal ions and increased the lipophilicity, which was conducive to the penetration of the complex into the lipid layer of the cell membrane. Recently, Nirmalya Bandyopadhyay et al. [59] synthesized a 2-hydroxylnaphthylhydrazone ligand with 3-substituted 2-ketoxime butane as a side chain using THF (tetrahydrofuran) as a solvent and further obtained a new four-coordinated Pd(II) metal complex for the first time (Figure 9). Structural characterization studies, including X-ray single-crystal diffraction analysis, showed that the central Pd(II) of the complex was in the “*N*/*N*/*O*/*Cl*−” coordination environment of a distorted planar square, in which Cl acted as the potential leaving group. Electrochemical study of this Pd(II) complex with Ag/AgCl as a reference electrode in DMF solution revealed an irreversible Pd(II)/Pd(I) redox property at ~0.646 V. The results of in vitro antibacterial tests and SEM (scanning electron microscopy) observations showed that this complex had good antibacterial activity against a variety of typical pathogens and fungi, while the naphthylhydrazone ligand had no antibacterial activity. At the concentration of 100 μg/mL, the inhibition zone of the complex to each strain ranged from 11 to 26 mm, and the MIC values were between 100 and 200 μg/mL.

Similarly, the corresponding Pd(II) and Pt(II) complexes were simultaneously synthesized when the benzenesulfyl group was set as the side chain of 2-hydroxylnaphthylhydrazone, as previously reported by H. Güzin Aslan et al. [47]. The results showed that both the Pd(II) and Pt(II) complexes showed good antibacterial activities against all of the tested Gram-negative and Gram-positive bacteria, which were also higher than those of the Ni(II)/Cu(II)/Co(II) complexes reported at the same time. It can be seen that different metal ions combine with the same naphthylhydrazone ligand and play different synergistic effects, thus affecting the complex’s level of antibacterial activity. This is also one of the attractive features prompting discussions of the crucial role of metals in medicine.

**Figure 10:**
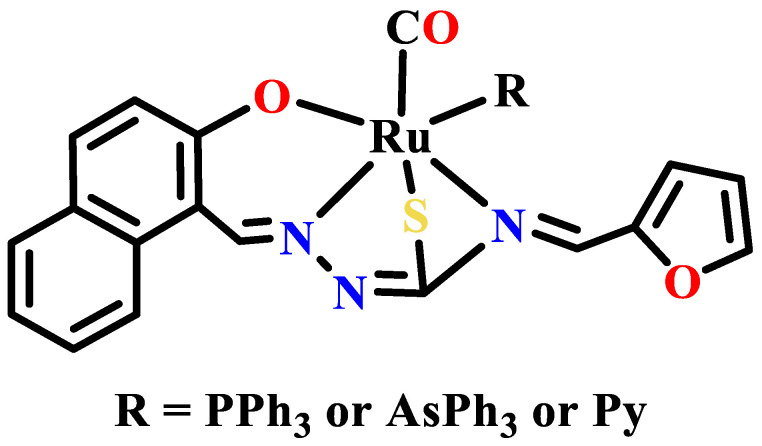
Three ruthenium(II) complexes of naphthalenehydrazone with *α*-furfuraldehyde amidyl thiourea.

G. Raja et al. [60] reported three ruthenium(II) complexes of 2-hydroxylnaphthalenehydrazone (those of phenylhydrazone were also involved) with *α*-furfuraldehyde amidyl thiourea as a side chain. Among these complexes, the new naphthylhydrazone ligand coordinated to Ru(II) in an *O*/*N*/*S*/*N*-tetradentate chelation mode, while the C=O group and PPh_3_/AsPh_3_/Py occupied the other two positions of the octahedral coordination configuration, as shown below in Figure 10. The results of the antibacterial activities indicated that for the ligand, the inhibition zones for *S. aureus* and *E. coli* had the ranges of 12~25 mm and 3~21 mm, respectively. However, the inhibition zones of the three Ru(II) complexes at the same concentration did not show obvious advantages. *E. coli* was more sensitive to the complexes than *S. aureus*, implying that the chelating coordination of this ligand with Ru(II) was beneficial to improving its transmembrane capacity to G− bacteria.

**Figure 11:**
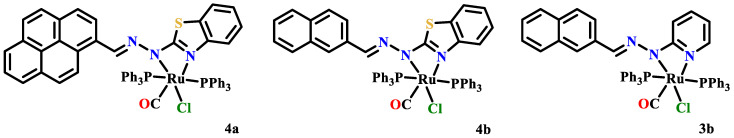
The ruthenium (II) complexes of pyrene/naphthyl hydrazone with benzothiazole or pyridine as a side chain.

The similar coordination pattern for the ruthenium(II) complex of naphthylhydrazone was reported by Soumitra Dinda et al. [61]. The naphthylhydrazone ligand in this work used benzothiazole or pyridine as the side chain, but this naphthylhydrazone ligand did not contain 2-OH, something that is relatively rare among existing studies (Figure 11). It was confirmed by X-ray single-crystal diffraction analysis that the ligands of these complexes were all electron donors to Ru(II) through *N*,*N*′-bidentate chelation, but the coordination N atom in this work was not from azomethine-N, but was instead from the deprotonated -NH groups of the hydrazone, which was attributed to the more flexible acylhydrazone. The C=O group and Cl^−^ combined with the two N atoms to form the equatorial plane of the Ru(II) coordination octahedron, while the two PPh_3_ occupied the axial position. Multiple transitions in the electron spectra of complexes can also be explained by the time-dependent density functional theory (TDDFT). The antibacterial activities of the Ru(II) complexes were tested in vitro for up to 12 typical pathogens. The results showed that the tested fungi (*Malassezia*, *Alternaria*, *Exserohilum*, and *Aspergillus*) were not particularly sensitive to the complexes. Interestingly, however, the tested bacteria were sensitive to both complexes, and complex **4** was the most active, while the side chain of its corresponding naphthylhydrazone ligand was benzothiazole. It appears an aromantic ring plane with a larger area helps to increase the antibacterial activity of the metal complexes of naphthylhydrazone, which is consistent with previous findings.

**Figure 12:**
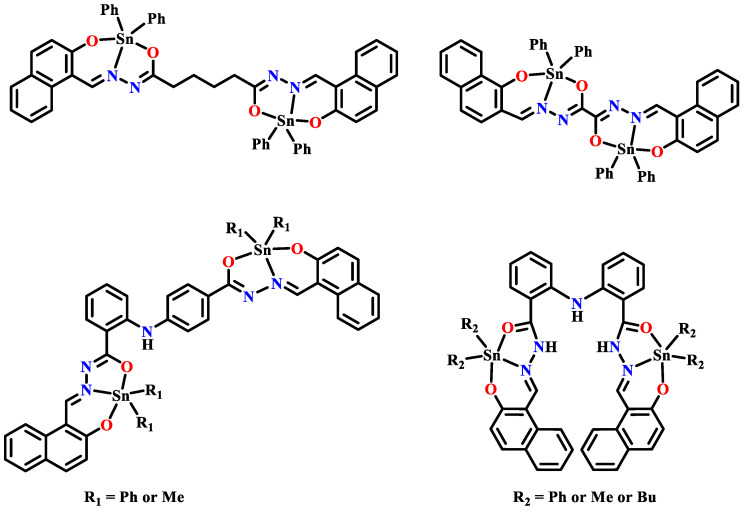
Isobinuclear organotin (IV) complexes based on dinaphthylhydrazone ligands.

In 2013, Tahereh Sedaghat’s group reported the synthesis of a series of isobinuclear organotin(IV) complexes based on dinaphthylhydrazone ligands (diphenylhydrazone ligands were also involved), which were linked by hexyldiamide, 2′/2″- (or 2′/4″-)diamide diphenylamine (Figure 12) [62,63,64]. The structures of these new compounds were characterized by IR, elemental analysis, and X-ray single-crystal diffraction analysis. In the above complexes, each dihydrazone acted as a tetra-base ligand in the form of enol, and tridentate coordinated with each central Sn(IV) via phenol-O, azomethine-N, and enol-O. Each Sn(IV) had a five-coordinated geometry, with the other two positions occupied by a pair of methyl/butyl/phenyl groups to form a symmetric binuclear complex. The in vitro antimicrobial activities of some of the ligands and all of the complexes towards the typical G+ bacteria (*B. subtilis* and *S. aureus*) and G− bacteria (*E. coli* and *P. aeruginosa*) were tested and compared with the positive control drugs (vancomycin and nalidixic acid) [62,63]. The results showed that most of the complexes showed stronger antibacterial activity than the ligands. However, it is noteworthy that the dihydrazone ligand linked by the aromatic diphenylamine as well as its Sn(IV) complex showed no significant inhibitory activity against these tested strains [64].

**Figure 13:**
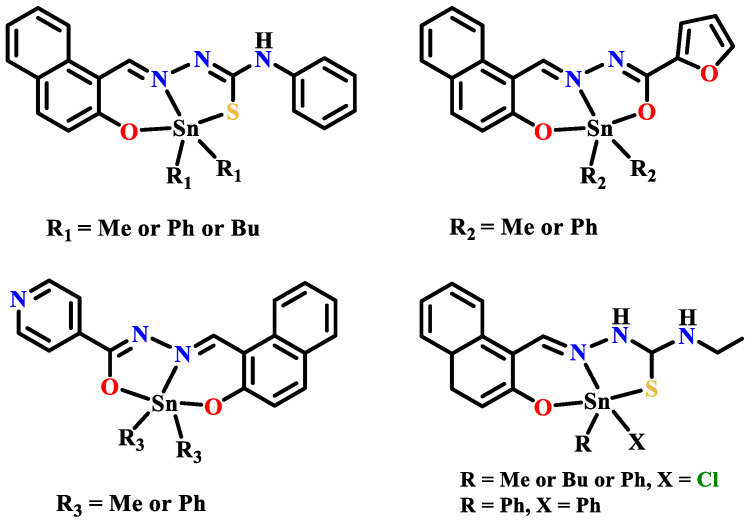
A series of mononuclear organotin(IV) complexes of 2-hydroxylnaphthylhydrazone.

In addition to the Sn(IV) complexes of bishydrazone, Sedaghat’s group [65,66,67] also reported a series of mononuclear organotin (IV) complexes of 2-hydroxylnaphthylhydrazone, in which the side chain of the hydrazone was phenylthiocarbazone, resulting in a dianionic ligand undergoing tridentate chelation by phenol-O, azomethine-N, and thiol-S. The coordination number of these Sn(IV) complexes was also five, and the other two sites were similarly occupied by methyl/phenyl groups (Figure 13) [65]. By comparing the antibacterial activities of these compounds in vitro, it was found that the naphthylhydrazone ligand showed weak antibacterial activity (except for the 5′-Br-phenylhydrazone), while the corresponding organotin(IV) complexes showed stronger antibacterial activity overall. In addition, the antibacterial activity of the complexes with different methyl/phenyl groups was also different, suggesting that the antibacterial activity of the complex depends on both the naphthylhydrazone ligand and the organic groups directly coordinated to Sn(IV). On the other hand, when 2-furanformamide [66] or isoniazid [67] was substituted for phenylthiosemicarbazide on the side chain of a naphthylhydrazone, the corresponding Sn(IV) complex exhibited different antibacterial activity. In terms of the ligand, the activity of the naphthylhydrazone bearing a 2-furanformamide side chain was higher than that of 5′-Br-phenylhydrazone. In contrast, the corresponding phenyl-coordinated organotin(IV) complex was more active than the methyl-coordinated one [66]. However, for the Sn(IV) complex of naphthylhydrazone with isoniazid as a side chain, its overall antibacterial activity was significantly decreased, and only showed weak antibacterial activity for the G+ bacteria (*B. subtilis* and *S. aureus*). For the two G− strains (*E. coli* and *P. aeruginosa*), however, the antibacterial activity was lost, even when the concentration was increased to 40 mg/mL [67]. Prior to this, in 2006, M. A. Salam et al. [68] reported a 2-hydroxylhydrazone ligand with a *N*-ethyl-substituted thiosemicarbazone as the side chain (Figure 13) as well as some of its mononuclear organotin (IV) complexes. In addition to the *O*/*N*/*S*-tridentate hydrazone, Cl^−^ and the methyl/butyl/phenyl group also coordinated with Sn(IV) to form five-coordinated organotin (IV) complexes. The in vitro antibacterial activity results showed that these organotin (IV) complexes also had good antibacterial activity against the tested typical pathogens, but only the Sn(IV) complex with diphenyl coordination had similar antibacterial activity to the positive control drug, ciprofloxacin, and the other compounds had lower antibacterial activity than ciprofloxacin. The inhibition zones of the butyl-Sn(IV) and methyl-Sn(IV) complexes ranged from 22.5 to 25.8 mm, suggesting that the organic groups that had coordinated to the central Sn(IV) had a significant effect on their biological activity, and this enhanced activity might be due to the chelation of the naphthylhydrazone ligand with Sn(IV), increasing the lipophilicity required for these Sn(IV) complexes to penetrate the bacterial membranes.

### 2.2. Antibacterial Metal Complexes of the Other Polycyclic Aromatic Hydrazones

**Figure 14:**
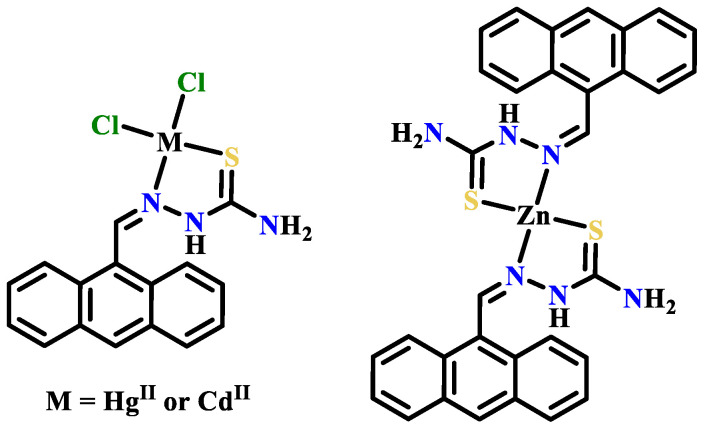
Zinc(II), cadmium(II), and mercury(II) complexes based on novel anthrahydrazone ligand with thiourea side chain.

There are limited studies on the antibacterial activities of polycyclic aromatic hydrazones with more than two rings. Previously, Floyd A. Beckford et al. [69,70,71] studied anthrahydrazone-based compounds. In 2006, they synthesized two novel anthrahydrazone and benzoanthrahydrazone ligands bearing a thiourea side chain via the condensation reaction of thiosemicarbazide or its *N*-ethyl derivatives with 9-anthracaldehyde or benzoanthrquinone, respectively, and then synthesized the corresponding heavy metal complexes, examples of which include zinc(II), cadmium(II), and mercury(II) (Figure 14). In the metal complex of anthrahydrazone with thiourea as a side chain, the ligand is chelated with Zn(II)/Cd(II)/Hg(II) by thiourea-S and azomethine-N. The stoichiometric ratio of metal to the ligand of the zinc(II) complex is 1:2, and that of the Hg(II) and Cd(II) complexes is 1:1 [71]. In vitro antibacterial activity tests showed that all complexes had certain inhibitory effects on both the G+ strains (*B. cereus* and *S. aureus*) and the G− strains (*P. vulgaris*, *E. faecalis* and *S. typhimurium*), and their activity was generally higher than that of the ligand. Among them, the Cd(II) and Hg(II) complexes were the most sensitive to *B. cereus* and *P. vulgaris*, respectively, with inhibition zones of 6 mm at 0.001 M. However, the ligand demonstrated no activity towards the G+ strains, and the inhibition zones towards G− strains were also ≦3 mm [71].

**Figure 15:**
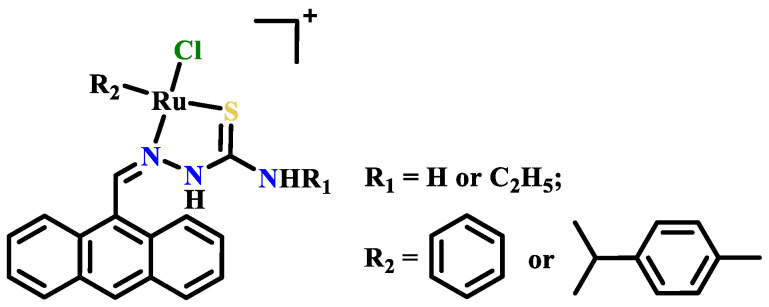
The organo-Ru(II) complexes of anthrahydrazone ligand with thiourea/ethylthiourea side chain.

They also reported on the corresponding ruthenium(II) complexes and tested the antibacterial activity of each compound against several typical pathogens using the disk diffusion and chemo-luminescence methods (Figure 15). The results showed that the activity of the two anthrahydrazone ligands was superior to that of the Ru(II) complexes. Among them, the anthrahydrazone ligand with thiourea as a side chain had the highest antibacterial activity. The antibacterial zone of *E. faecalis* in particular reached 10 mm (0.001 M), which was much higher than that of the benzoanthrahydrazone ligand and two Ru(II) complexes and more than twice that of the positive control drug, chloramphenicol. Unfortunately, each Ru(II) complex showed no significant antibacterial activity, indicating that not all hydrazone ligands can improve their activity after coordination with metal ions, and the role of metal ions was thus more interesting to explore further [69]. Subsequently, in 2011, they synthesized another organic ruthenium(II) complex with *p*-methyl isopropylbenzene as the co-ligand [70], which not only showed antitumor activity, but also showed a significant bacteriostatic effect against G+ bacteria, especially against *B. cereus*, with a MIC value as low as 5 μM. The MIC value of this Ru(II) complex against *E. faecalis* also reached 20 μM, although it was still inactive against G− bacteria at the maximum concentration tested [70].

**Figure 16:**
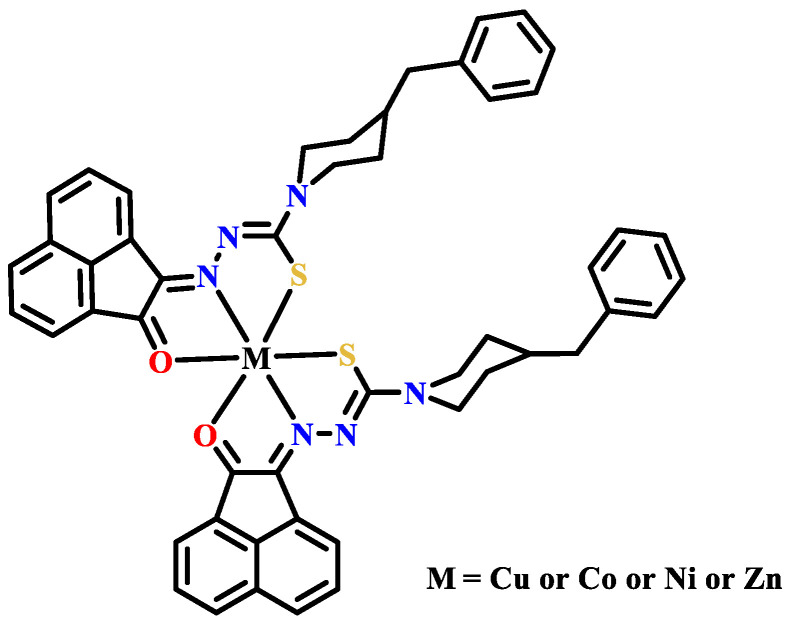
The four Co(II)/Ni(II)/Cu(II)/Zn(II) complexes based on 1-acenaphthenone hydrazone ligand with 3-(4-benzylpiperidyl) thiourea side chain.

Recently, Santosh Kumar et al. [72] also reported a novel 1-acenaphthenone hydrazone ligand with 3-(4-benzylpiperidyl) thiourea as side chain and further synthesized four new transition metal complexes based on this ligand (Figure 16). The four Co(II)/Ni(II)/Cu(II)/Zn(II) complexes were all hexagonal octahedral complexes, in which two acenaphthenone hydrazone ligands tridentate coordinated in *cis*- form via carbonyl-O, azomethine-N and thiol-S. The measured room-temperature magnetic moments of the Co(II) and Ni(II) complexes indicated that they were in a high spin state and adapted a distorted octahedral configuration. In vitro antibacterial test results showed that all of the complexes (<50 μg/mL) had better antibacterial activity against the same pathogen than the ligand (>100 μg/mL). Especially for the Ni(II) complex, although its activity was still lower than gentamicin (1–2 μg/mL), it showed the strongest inhibitory activity against all of the tested pathogens with MIC values of 5~10 μg/mL. The interaction patterns of the Ni(II) complex with *E. coli* and *B. subtilis* were also examined using a scanning electron microscope (SEM). The results showed that the cell walls of bacteria treated with Ni(II) complex were damaged. Therefore, it was speculated that the cell wall damage was one of the key mechanisms for the complex to effectively inhibit the proliferation of the pathogens.

## 3. The Anticancer Activity Study

### 3.1. Anticancer Metal Complexes of Naphthylhydrazone

#### 3.1.1. The First Transition Metal Complexes with Anticancer Activities

Vanadium is one of the important essential elements for life. The electronic structure of its valence shell (3d^3^4s^2^) gives it diverse valence states. Vanadium plays an important role in living organisms, such as in cells and physiological media, in which vanadium mainly exists in the complex form of +4-charged VO^2+^ (oxyvanadium cation) and +-charged VO_2_^+^ or polyvanadate. According to the principle of similarity of ions, vanadium complexes have a wide range of biological activities that can regulate the redox process in cells and interfere with the metabolism of calcium, iron, and phosphate in vivo [73]. At the same time, vanadium complexes also have a variety of pharmacological activities, such as hypoglycemic [74] and hypolipidemic activities [75]. However, the research on vanadium complexes is still mainly focused on its antidiabetes and anticancer activities, especially the former, which is a large area of concern. Although vanadium complexes have been found to have a significant tumor prevention effect in the long term, there are still very few reports about the vanadium complexes of naphthylhydrazone [76,77].

**Figure 17:**
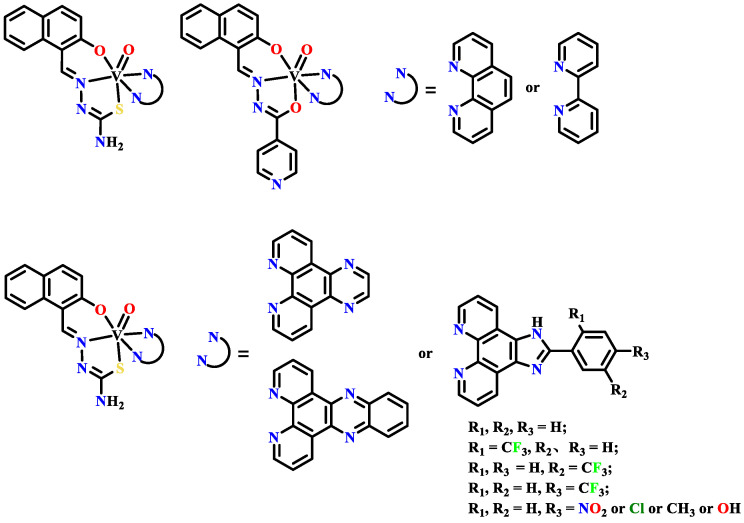
Several vanadium (IV) complexes of naphthylhydrazone ligand with thiourea or isoniazide side chains.

In 2012, Jiazheng Lu et al. [76] synthesized four hexa-coordinated VO complexes of the *O*/*N*/*S* tridentate 2-hydroxylnaphthylhydrazone ligand (including phenylhydrazone) by using thiourea as a side chain and aided by various *N*,*N*′-bidentate co-ligands such as *o*-phenanthroline and bipyridine (Figure 17). Their binding properties to DNA were studied by spectroscopic analysis, viscosity determination, and thermal denaturation analysis. The results showed that four vanadium(IV) complexes could intercalate with DNA and cleave plasmid pBR322 DNA effectively. Meanwhile, these V(IV) complexes showed significant cytotoxic activity against two tested cancer cell lines, myeloma (Ag8.653) and glioma (U251), and were significantly superior to those V(IV) complexes of phenylhydrazone as the same series. In particular, for the V(IV) complex with *o*-phenanthroline as a co-ligand, the half maximal inhibitory concentration (IC_50_) values were towards Ag8.653, and U251 reached 4 nM and 7 nM, showing extremely high anticancer activity in vitro. Subsequently, this group [78] replaced the side chain group with isoniazide and synthesized a series of vanadium(IV) complexes with similar structure under the same conditions. The results showed that these V(IV) complexes had the ability to scavenge ·OH radicals under certain conditions and had good inhibitory activity against MCF-7, SH-SY5Y, and SK-N-SH cancer cell lines, especially against neuroblastoma cells. Additionally, the complexes with phenanthroline as a co-ligand showed the highest activity towards two neuroblastoma cell lines, SH-SY5Y and SK-N-SH, with IC_50_ values of 3.95 μM and 1.21 μM, respectively. Similar to the complexes reported above, the V(IV) complex of naphthylhydrazone with isoniazide as a side chain showed higher anticancer activity than the phenylhydrazone complex of the same series, and the complex with *o*-phenanthroline as a co-ligand also demonstrated the most significant anticancer activity. According to the SAR of the V(IV) complexes of these two kinds of naphthylhydrazone, the anticancer activity of *O*/*N*/*S*-tridentate VO complexes may be higher than that of *O*/*N*/*O*-tridentate VO complexes.

This research group [77,79,80] as well as Yin-Liang Bai et al. [81] selected a naphthylhydrazone ligand with thiourea as the side chain that showed higher anticancer activity and further introduced phenylimidazolphenanthroline and pyrazinphenanthroline as new co-ligands (Figure 17). They also continued to synthesize V(IV) complexes with a similar coordination structure. The results showed that the new V(IV) complex could also effectively cleave DNA and showed good anticancer activity, and the complex with higher activity could block the tumor cell cycle and induce apoptosis. These experimental results are helpful to further explore and understand oxyvanadium(IV) complexes based on naphthylhydrazone as potential metal-based anticancer agents. The results showed that the V(IV) complexes bearing phenylimidazolphenanthroline were superior to those bearing pyrazinphenanthroline, the anticancer activities of which were 2~28 times those of the latter. Specifically, the anticancer activity of the V(IV) complexes of phenylimidazolphenanthroline was closely related to the substituents on the benzene ring (such as CF_3_, Cl, NO_2_, etc.), among which the corresponding complexes with benzene with *para*-CF_3_ had significantly better anticancer activity than those substituted on the *ortho*- or *meso*- positions.

**Figure 18:**
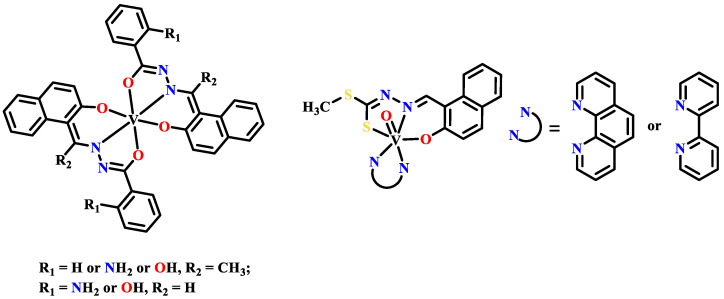
Several vanadium (IV) complexes reported by Rupam Dinda’s group.

The group of Rupam Dinda [82,83,84] reported the vanadium complex of 2-hydroxynaphthylhydrazone using benzoylhydrazine instead of isoniazide as a side chain, in which the -NH_2_ or -OH introduced to the 2-position of benzene ring was used for SAR comparison (Figure 18). In each complex, two naphthylhydrazone ligands were coordinated with the center V(IV) in an *O*/*N*/*O*-tridentate chelation mode to form a hexagonal octahedral geometry. Therefore, the complex was of the non-VO type but could be stable in both the solid and solution states, which is very rare in a non-VO(IV) complex. All of these complexes showed in vitro anticancer activity and had good insulin-fitting activity [83]. Some complexes were found to undergo a valency transition in aqueous solution, and their interaction sites with ubiquitin and lysozyme proteins were found by molecular docking [84]. Recently, they further synthesized three new VO(IV) complexes with a distorted octahedral configuration by introducing bipyridine and *o*-phenanthroline as the co-ligand and using *S*-methyl dithiourea as the side chain of the naphthylhydrazone ligand (including phenylhydrazone) (Figure 18) [85]. All of the complexes could bind to ct-DNA and human serum albumin (HSA) to different degrees. The binding constants to the DNA of the V(IV) complexes of naphthylhydrazone, with *o*-phenantholine and bipyridine as the co-ligand were 8.24 × 10^3^ and 3.66 × 10^4^ M^−1^, and to HSA, they were 3.87 × 10^6^ and 9.75 × 10^6^ M^−1^, respectively. This suggests that different co-ligands may affect the binding ability of the corresponding complexes with HSA and DNA. Furthermore, these VO(IV) complexes also inhibited the proliferation of human cervical cancer cells (HeLa) and mouse embryonic fibroblasts (NIH-3T3).

**Figure 19:**
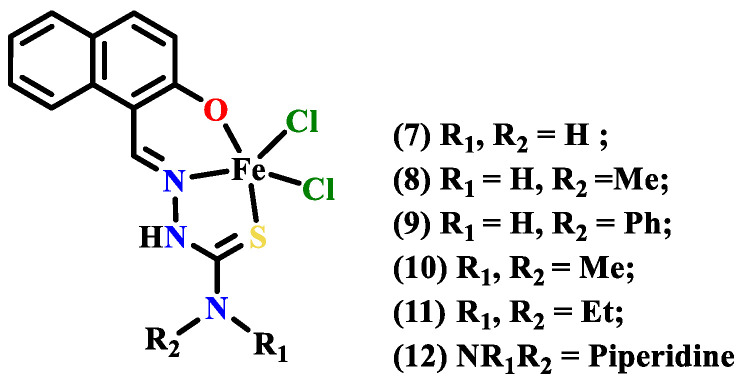
Six new Fe(III) complexes of 2-hydroxylnaphthylhydrazone with *N*-substituted thiourea side chains.

In order to improve the selectivity, transport, and activity of iron-based anticancer agents, Jinxu Qi et al. [86] designed pro-drugs based on the *N*-donor residues of the human serum albumin (HSA) IIA subdomain as the carrier, in which six new Fe(III) complexes of 2-hydroxylnaphthylhydrazone with *N*-substituted thiourea as the side chain were set for the central drugs (Figure 19). They could bind with HSA to form the HSA complex as pro-drugs. The in vitro antitumor activity of the Fe(III) complexes encapsulated with HSA was significantly increased. The activity of the Fe(III) complex of 2-hydroxylnaphthylhydrazone with *N*-piperidinium thiourea as the side chain (complex **12**) was the highest. In vivo antitumor experiment results showed that complex **12** and the HSA-**12** complex could inhibit the proliferation of liver cancer, and the HSA-**12** complex had stronger targeting and therapeutic effects than **12**. Due to strong binding to the IIA subdomain of HSA, certain properties of the Fe(III) complexes were improved, including delivery efficiency, antitumor activity, and selectivity. Compared with the Fe(III) complexes alone, the HSA complexes had better tolerability, higher drug accumulation in tumor tissues, and lower toxicity, suggesting better antitumor effects with fewer side effects. These results suggest that the intravenous administration of the Fe(III) complex of 2-hydroxylnaphthylhydrazone using HSA as a pro-drug carrier might be a promising approach for targeted tumor therapy.

Copper is also one of the essential trace elements in the life process and maintains the redox cycling ability in biological systems. This is because of the various coordination modes and sensitive redox states of copper that occur when researchers combine it with organic ligands with potential pharmacological activity and try to maximize the potential pharmacological activity of both.

**Figure 20:**
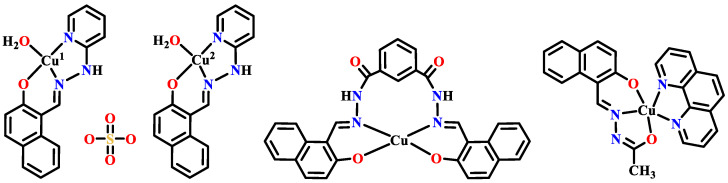
Several different types of Cu(II) complexes of 2-hydroxylnaphthylhydrazone ligands.

Soma Mukherjee et al. [87] synthesized a 2-hydroxylnaphthylhydrazone ligand with a 2-pyridinyl group as a side chain and achieved a new cationic Cu(II) complex through the coordination reaction of this hydrazone ligand to copper(II) by the tridentate phenol-O, azomethine-N, and pyridine-N atoms along with the existing H_2_O and SO_4_^2−^ as the counter ions. Thus, the complex had a four-coordinate and slightly distorted planar quadrilateral geometry. The binding mode of this Cu(II) complex with DNA was studied by UV—Vis spectroscopy, fluorescence emission spectroscopy, viscosity experiments, and thermal denaturation analysis. In vitro testing of the anticancer activity showed that the Cu(II) complex had antiproliferative activity against HeLa cells and could block the cell cycle at the G2/M phase but could not induce apoptosis, showing its unique anticancer mechanism. S.M. Pradeepa et al. [88] designed a new *m*-benzenebisnaphthylhydrazone using *m*-benzenebisacylhydrazine as a bridging group, as shown in Figure 20 below. Three transition metal complexes (Co, Ni, Cu) were synthesized as potential new photosensitizers for photodynamic therapy. The central metal of each complex was tetradentate-chelated with the two azomethine-N atoms and two 2-phenol-O atoms of this bisnaphthylhydrazone ligand. The Cu(II) complex was determined to interact with DNA, mainly by the surface-groove-binding mode, and could cleave supercoiled plasmid DNA under 365 nm of ultraviolet light, which significantly enhanced the cytotoxic effect of the complex towards A549 lung cancer cells. Additionally, the proliferation inhibition rate of the Cu(II) complex on the cancer cells was obviously higher than those of the bisnaphthylhydrazone ligand and the other two complexes. It was speculated that the singlet oxygen produced by the Cu(II) complex under these conditions played an important role in DNA photolysis. Therefore, it showed significant cytotoxic activity and was regarded as a potential PDT (photodynamic therapy) reagent. Iran Sheikhshoaie et al. [89] synthesized a ternary mixed copper(II) complex using a 2-hydroxynaphthylhydrazone ligand with an acetamide side chain and *o*-phenanthrene as the co-ligand. X-ray single-crystal diffraction analysis showed that the central Cu(II) complex had a five-coordinate tetragonal pyramidal geometry that was coordinated with the *O*/*N*/*O*-tridentate naphthylhydrazone and *N*,*N*′-bidentate *o*-phenanthrene (Figure 20). The in vitro anticancer activity showed that the complex had strong growth inhibition on MCF-7 human lung cancer cell lines, much higher than the naphthylhydrazone ligand, *o*-phenanthroline, and Cu(NO_3_)_2_ alone.

**Figure 21:**
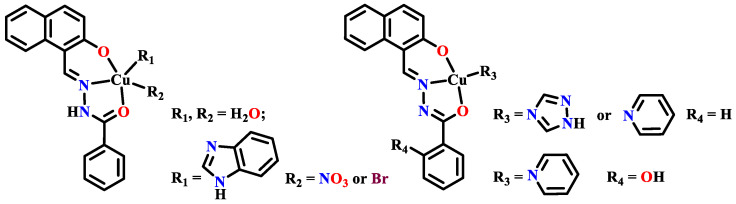
A series of Cu(II) complexes of naphthylhydrazone with benzamide derivatives as side chains together with different co-ligands for SAR studies, recently reported by F. Yang’s group.

In recent years, F. Yang’s group has also become interested in the metal complexes of 2-hydroxylnaphthylhydrazone [90,91,92]. They selected benzoamide and 2′-hydroxylbenzoamide as the side chains of naphthylhydrazone to synthesize the corresponding ligands and further selected imidazole, benzimidazole, pyridine, etc., as co-ligands to synthesize a series of copper(II) complexes with relatively high anticancer activity (Figure 21). Among them, several Cu(II) complexes of naphthylhydrazone along with the pyridine co-ligand could cleave DNA, block the cell cycle, and eventually induce cell apoptosis [91]. Those Cu(II) complexes with imidazole or benzimidazole co-ligands could combine with HSA to form an HSA-Cu complex. Similarly, the HSA-Cu complex showed higher cytotoxicity, could more efficiently arrest the cell cycle and lead to cell death through the reactive oxygen species (ROS)-mediated mitochondrial pathway in the tested cancer cells compared to the Cu(II) complex alone [92]. Comparing the anticancer activity of the Cu(II) complexes, the results indicated that the Cu(II) complexes with these *N*-containing heterocycle co-ligands (imidazole, benzimidazole, pyridine, and triazole) exhibited more significant antitumor activity. On the other hand, the Cu(II) complexes of 2-hydroxylnaphthylhydrazone with 2′-hydroxybenzoamide as a side chain had comparatively weaker activity, showing a certain structure–activity relationship.

**Figure 22:**
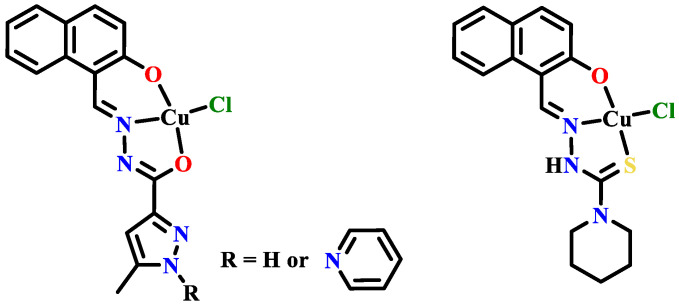
Different Cu(II) complexes of naphthylhydrazone with imidazolamide or piperidinyl thiourea as side chains.

Nádia Ribeiro et al. [93] recently reported two copper (II) complexes of 2-hydroxynaphthylhydrazone with imidazolamide as a side chain (Figure 22). The structural difference was determined by whether the 1-N atom of imidazolium is replaced by 2′-pyridine. Their IC_50_ values for MCF-7 cancer cells were almost the same and were about 2.6 μM. While for PC3 cancer cells, the pyridine-substituted one still had an IC_50_ value of about 2.6 μM, but that of the non-substituted one was 7.71 μM, which was weaker than the previous one. Both complexes could effectively bind with DNA and HSA. Experimental analysis showed that the mechanism of cell death of the tested PC3 cells caused by the complexes was not the apoptosis-mediated by the activation of caspases-3/7 but was instead mediated by changing the membrane potential of PC3 cells, thereby affecting the imbalance of physiological elements such as P, K, and Ca in cells. Yihong Wang’s group [94] also recently synthesized a 2-hydroxylnaphthylhydrazone ligand with piperidinyl thiourea as a side chain as well as its copper(II) complex (Figure 22), in which the naphthylhydrazone ligand was coordinated with Cu(II) in an *O*/*N*/*S*-tridentate chelation mode and formed a planar quadrilateral configuration with a Cl atom. In vitro anticancer screening towards four typical human cancer cell lines (HeLa, T-24, BEL-7404, and NCI-H460) indicated that the ligand had the significant characteristic of being a cytotoxic agent, with IC_50_ values ranging from 3 to 4 μM. The Cu(II) complex showed higher activity with IC_50_ values of 70–90 nM. Compared with the ligand, the in vitro activity of the Cu(II) complex against all of the tested cancer cell lines was more than 40 times higher, and it could significantly induce the apoptosis of HeLa cells at lower concentrations. Studies on the apoptosis mechanism showed that this Cu(II) complex could effectively catalyze the production of ROS via hydrogen peroxide, and the excessive ROS further led to the dysfunction of mitochondrial membrane potential and promoted the release of mitochondrial apoptotic factors.

**Figure 23:**
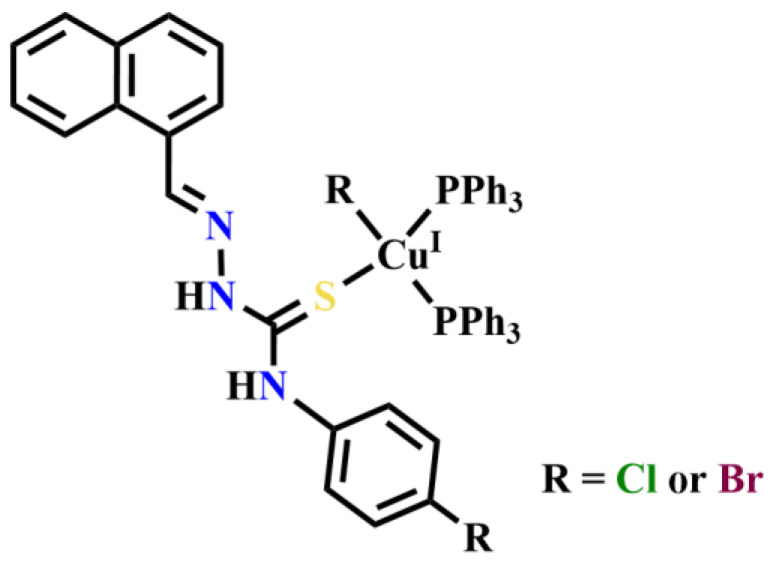
A pair of Cu(I) complexes of the mono-coordinated naphthylhydrazone by S atoms from the *p*-halophenyl thiourea side chain, reported by Rupam Dinda’s group.

On the other hand, Rupam Dinda’s group also synthesized two specific copper(I) complexes of naphthylhydrazone (Figure 23) [95]. Less commonly, this naphthylhydrazone ligand does not contain a 2-hydroxyl group, while the side chain group was selected to be (*p*-X-phenyl)thiourea (X = Cl or Br). Therefore, this naphthylhydrazone only coordinated with Cu(I) through S atoms, while another two PPh_3_ and one halogen atom (the same as the X atom on the side chain) coordinated with the center Cu(I), forming a four-coordinated tetrahedral configuration. The results showed that the complex could bind to ct-DNA via the groove-binding mode and showed photo-induced DNA cleavage activity, which could have been achieved through singlet oxygen and hydroxyl radical pathways. Both the two Cu(I) complexes had certain and similar in vitro growth-inhibitory activity against HeLa cancer cells, but compared with the reported Cu(II) complexes of naphthylhydrazone above, the activity difference was up to several hundred times, and the IC_50_ value was only approximately 30 μM. The significant difference in activity may be related to the coordination mode of the two kinds of copper complexes. We think the large steric hindrance of the two PPh_3_ groups, which have binding affinity to the softer Cu(I), might significantly block the copper center from exerting its action mechanism which may affect the anticancer activity of these Cu(I) complexes. Moreover, the lack of rigidity of the Cu(I) complexes might also lead to a decrease in its DNA binding ability.

Very recently, this group [96] also reported a new binuclear zinc(II) complex of 2-hydroxynaphthylhydrazone with *p*-methoxyphenyl thiourea as a side chain, in which each central Zn(II) had a five-coordinated pyramidal geometry. Both naphthylhydrazone ligands coordinated with Zn(II) via *O*/*N*/*S*-tridentate chelation, while two phenolic-O atoms acted as bridging atoms to link the two Zn(II) atoms. Each Zn(II) also had a DMSO molecule involved in coordination, occupying the conical position of the pyramid, thus forming the symmetric binuclear Zn(II) complex. This special Zn(II) complex showed strong binding ability to both DNA and HSA. In addition, it had obvious growth inhibitory activity against a human cervical cancer cell line (HeLa) and a colon cancer cell line (HT-29), with IC_50_ values of 16.26 μM and 18.32 μM, respectively, and could further induce the apoptosis of cancer cells. In addition, the phosphatase activity of this Zn(II) complex was detected using bis(2,4-dinitrophenyl) phosphoric acid (BDNPP) as a substrate, but unfortunately, no significant catalytic activity of the complex was found.

**Figure 24:**
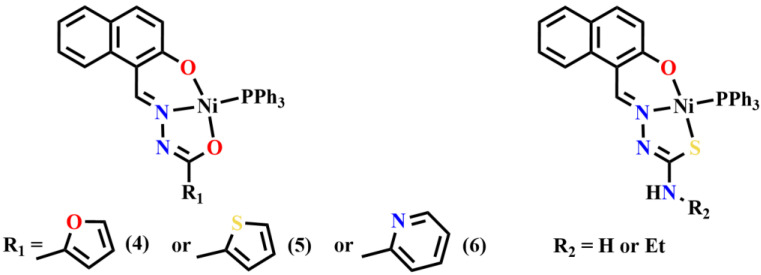
A series of Ni(II) complexes of naphthylhydrazone with furan/thiofuran/pyridine amides or thiourea/ethylthiourea as side chains.

Anticancer nickel(II) complexes of naphthylhydrazone have also been explored and studied in the past ten years. Paramasivam Krishnamoorthy et al. [97] started related research on this topic in 2012. They designed three 2-hydroxylnaphthylhydrazone ligands with furan, thiofuran, and pyridine amides as side chains and then synthesized three new corresponding nickel(II) complexes (Figure 24). In addition to the *O*/*N*/*O*-ternary-chelated naphthylhydrazone ligands, the fourth coordination position was occupied by PPh_3_, forming a planar quadrilateral configuration. The in vitro anticancer test results showed that the three Ni(II) complexes had certain inhibitory effects on the proliferation of the three kinds of cancer cells, especially A431 cells, showing stronger proliferation than in the HeLa and HepG2 cell lines, while neither the ligand nor nickel salt demonstrated obvious inhibitory activity. On the other hand, almost none of these Ni(II) complexes were toxic to the normal cell line (NIH3T3), suggesting that the complexes had some toxic selectivity to cancer cells. In addition, these Ni(II) complexes can effectively bind to DNA and HSA, which provides some insight into the action mechanism for their anticancer activity. However, Ni(II) complex **6**, which contains a pyridine side chain, has the strongest binding ability to DNA and HSA, showing a certain structural—activity relationship. Furthermore, the anticancer activity of complex **6** was also more significant in vitro. Therefore, for SAR, it was primarily speculated by the authors that the size of the heterocyclic ring (from a five-membered ring to six-membered ring) and the electronegativity of the heterocyclic atoms in the side chain of naphthylhydrazone could affect the anticancer activity of the complex. However, compared with the Cu(II) complexes of the similar naphthylhydrazones reported previously, the anticancer activities of these Ni(II) complexes are obviously weaker.

At about the same time, Sayanti Datta [98] and R. Prabhakaran et al. [99] also synthesized corresponding Ni(II) complexes using a 2-hydroxynaphthylhydrazone ligand with thiourea or ethylthiourea as the side chain (Figure 24). Among them, the Ni(II) complex with pyridine as the co-ligand showed higher activity, with an IC_50_ value of 7.6 μM against the MCF-7 human breast cancer cell line. The complex could induce apoptosis to a certain extent (about 6.8%) in MCF-7 cells at the level of 10 μM, but this ratio of apoptosis was actually not high, suggesting that apoptosis induction was not the primary anticancer mechanism of this Ni(II) complex. In addition, it also showed that the Ni(II) complex was also an effective catalyst for the Heck-type C-C coupling reaction [98]. Experimental studies on the other two Ni(II) complexes (thiourea and *N*-ethylthiourea as side chains) showed that they have certain antioxidant activities that could effectively eliminate 1,1-diphenyl-2-picrylhydrazine (DPPH) and also overcome the resistance of A549 cells to cisplatin [99].

**Figure 25:**
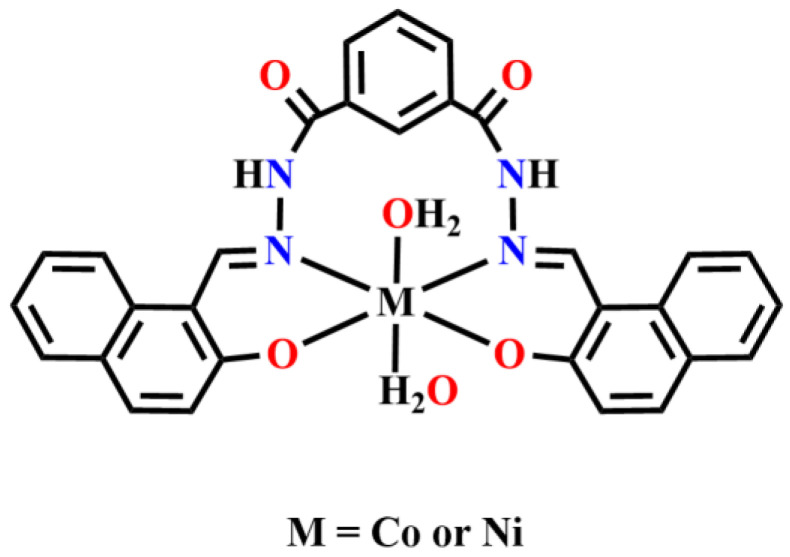
The six-coordinated Co(II)/Ni(II) complexes of *m*-benzenebisnaphthylhydrazone, with two H_2_O molecules as the axial ligand.

We have also introduced three kinds of transition metal complexes of *m*-benzenebisnaphthylhydrazone, previously developed by S.M. Pradeepa et al. [88]. In addition to the four-coordinated copper(II) complex, the other two kinds of Co(II) and Ni(II) complexes had a six-coordinated octahedral configuration, while two axial positions of the octahedron were occupied by two H_2_O molecules, as indicated in Figure 25. They could also be used as new photosensitizers for PDT. Unlike the previous Cu(II) complex, the Co(II) and Ni(II) complexes bound to DNA in a covalent manner, and the cytotoxicity of the two complexes towards A549 was lower than that of the Cu(II) complex, suggesting that different metal centers may significantly affect the anticancer activity of the corresponding metal complexes.

**Figure 26:**
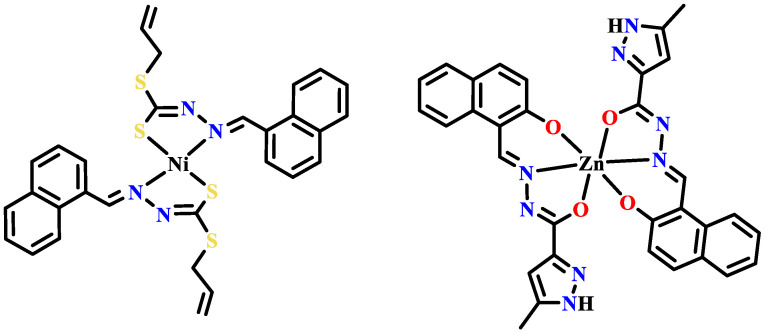
The symmetric Ni(II) and Zn(II) complexes coordinated by the same two naphthylhydrazone ligands with *S*-allyldithiocarbazate or methylimidazolamide as side chains.

Nanjan Nanjundan et al. [100] reported the design and synthesis of a naphthylhydrazone (phenylhydrazone was also involved) with *S*-allyldithiocarbazate as a side chain, and the corresponding nickel(II) complex was further obtained (Figure 26). Since the corresponding naphthylhydrazone ligand did not bear 2-OH, the Ni(II) complex was four-coordinated by two naphthylhydrazone ligands via imine-N and thiol-S to form a symmetrical Ni(II) complex with planar quadrilateral geometry. The binding constant of this naphthylhydrazone-Ni(II) complex with DNA was 3.54 × 10^4^ M^−1^, which was weaker than that of the corresponding phenylhydrazone-Ni(II) complex. On the contrary, the naphthylhydrazone-Ni(II) complex showed a higher binding ability with bovine albumin (BSA), with a Stern—Volmer binding constant, *K*_SV_, of 5.8 × 10^4^ M^−1^, which was stronger than that of the corresponding phenylhydrazone-Ni(II) complex. In addition, this Ni(II) complex showed moderate cytotoxicity to the Vero and HeLa cancer cell lines, with IC_50_ values of 65.51 and 25.13 µg/mL, respectively, slightly weaker than the corresponding phenylhydrazone-Ni(II) complex. Nádia Ribeiro et al. [101] recently synthesized an *O*/*N*/*O*-tridentate six-coordinated zinc(II) complex using the same two 2-hydroxy-naphthylhydrazones with methylimidazolamide as a side chain (Figure 26). The photophysical properties of the naphthylhydrazone ligands and Zn(II) complexes were studied by means of theoretical calculations. The results showed that the keto-tautomerism of the ligand under excitation was stabilized in the energy-accessible triplet states, but not in the Zn(II) complex. The binding ability of the Zn(II) complex with DNA was measured as a fluorescence probe. Compared to the Cu(II) complex with the same ligand [93], although the Cu(II) complex had stronger cytotoxicity (IC_50_ = 7.71 μM) to PC3 cells than the Zn(II) complex (IC_50_ = 35.2 μM) towards the MCF-7 cell line, the Zn(II) complex had the highest inhibitory activity, with an IC_50_ of 0.53 μM, which is rare in studies on the Zn(II) complexes of naphthylhydrazone, and was also much stronger than the Cu(II) complex of the same ligand, which also showed a comparatively high inhibitory activity (IC_50_ = 2.58 μM).

From these results, we are not arbitrarily inferring that the activities of the metal complexes of the *O*/*N*/*S*-tridentate naphthylhydrazone are higher than those of the *O*/*N*/*O*-tridentate ones. The influence of the central metals as well as the properties of the substituent groups of the side chains on the activity cannot be ignored. Therefore, it is necessary to explore and discuss the structure–activity relationship with regard to the anticancer activity in this research area.

#### 3.1.2. The Second and Third Transition Metal Complexes of Naphthylhydrazone with Anticancer Activities

**Figure 27:**
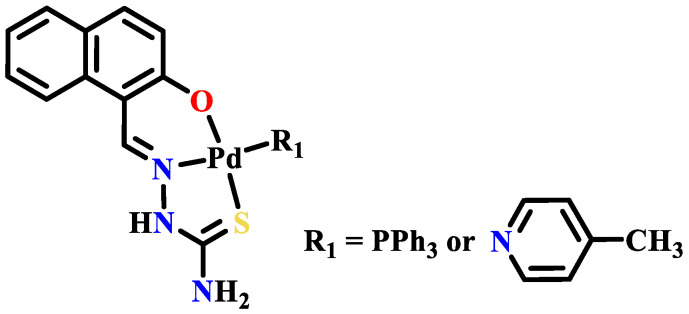
Two palladium(II) complexes of naphthylhydrazone with thiourea as a side chain along with PPh_3_ or *p*-methylpyridine as co-ligands.

In 2008, Sarmistha Halder et al. [102] reported two palladium (II) complexes of 2-hydroxynaphthylhydrazone with thiosemicarbazide as a side chain, comparing them to the corresponding Pd(II) complexes of 2-hydroxyphenylhydrazone (Figure 27). For the Pd(II) complexes with a typical four-coordinated mode, PPh_3_ and *p*-methylpyridine were selected as co-ligands to compare the SAR and also compared the *O*/*N*/*S*-tridentate naphthylhydrazone (or phenylhydrazone) ligand to the central Pd(II). In vitro anticancer activity tests showed that the Pd(II) complex of naphthylhydrazone with PPh_3_ as a co-ligand had almost no inhibitory activity against HL-60 and U-937 cancer cell lines, with IC_50_ values exceeding 200 μM, significantly higher than those of the corresponding Pd(II) complexes of phenylhydrazone (IC_50_ = 0.6~4.8 μM). However, the in vitro anticancer activity of the Pd(II) complexes of naphthylhydrazone and phenylhydrazone with *p*-methylpyridine as a co-ligand were very similar, with all of the IC_50_ values in the range of 6~7 μM, and did not show the influence of the number of rings of aromatic hydrazone on their anticancer activities. The results of this SAR are also rarely reported in related research work, showing an interesting contrast.

**Figure 28:**
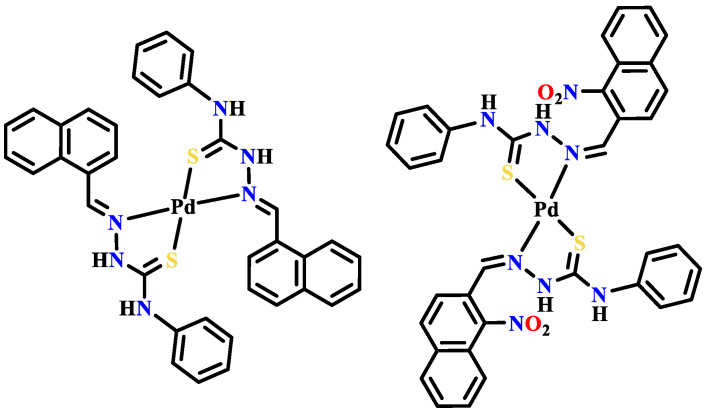
Two symmetric palladium (II) complexes of 1-naphthylhydrazone and 1-nitro-2-naphthylhydrazone, both with phenylthiourea as a side chain.

Wilfredo Hernández et al. [103] also carried out a very similar study using the same central metal, Pd(II), and the same phenylthiourea side chain (Figure 28). However, they focused on the influence of the positions of hydrazone and the substituted groups on the naphthalene ring over their anticancer activities. To this end, they designed two naphthylhydrazone ligands: one was a 1-naphthylhydrazone ligand of the same type previously reported but without 2-OH substitution and with a *N*-phenylthiourea side chain; the other was a rare reported ligand and 1-nitro-2-naphthylhydrazone with a *N*-phenylthiosemicarbazide side chain. Accordingly, they also obtained two new Pd(II) complexes and carried out subsequent screening and comparison with the Pd(II) complexes of three other non-naphthylhydrazone ligands. The results showed that the five Pd(II) complexes exhibited different anticancer activities in vitro. The IC_50_ values of these Pd(II) complexes against the six tested cancer cell lines (H460, DU145, MCF-7, M14, HT-29, and K562) ranged from 0.01 to 10 μM, with a difference of up to 1000 times, and the Pd(II) complex of the phenylhydrazone ligand was still the most active. In terms of naphthylhydrazone, the anticancer activity of the two ligands was relatively weak and were higher than 25 μM, with some even higher than 250 μM. However, the two corresponding naphthylhydrazone-Pd(II) complexes showed significantly higher inhibitory activity against all of these cancer cell lines, with IC_50_ values in the range of 0.65~2.39 μM, 12 times higher than that of the ligand. K562 was the most sensitive cell line for both Pd(II) complexes, with IC_50_ values of 1.84 and 0.65 μM. In contrast, the rare Pd(II) complex of the 1-nitro-2-naphthylhydrazone ligand showed higher activity, with overall activity 2~3 times that of the common complexes of 1-naphthylhydrazone. Therefore, it is necessary to strengthen and expand the research on metal complexes of the 2-naphthylhydrazone ligand to find more potential metal-based anticancer agents.

**Figure 29:**
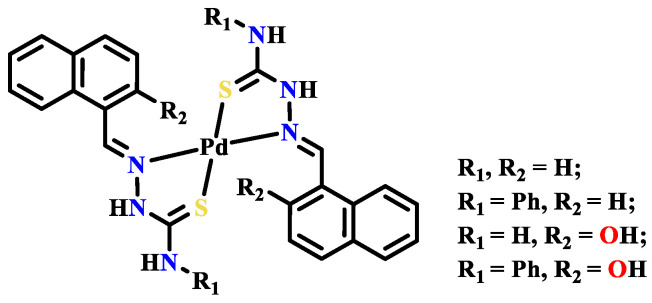
A series of palladium(II) complexes of naphthylhydrazone with *N*-substituted thiourea side chain.

In the above exploration, Wilfredo Hernández et al. found that a Pd(II) complex with 2-OH phenylhydrazone had the best activity, so they further attempted to synthesize the Pd(II) complexes with 2-hydroxylnaphthylhydrazone, and the side chain was still regulated by whether or not there was a phenyl group (Figure 29) [104]. Four new Pd(II) complexes were thus synthesized using the orthogonal method. The results of the in vitro cellular screening showed that the anticancer activity of all of the Pd(II) complexes was still higher than that of the ligands, indicating that the combination of Pd(II) and naphthylhydrazone formed a positive synergistic effect. Compared with the Pd(II) complexes, the overall activity of the Pd(II) complex decreased when the naphthylhydrazone (without 2-OH) side chain only contained phenyl. For 2-hydroxylnaphthylhydrazone, the presence of phenyl on the side chain also weakened the overall activity of the Pd(II) complex. The authors deduced that the presence of phenyl on the side chain of thiosemicarbazide might lead to the loss of the inhibitory activity against cancer cells due to its high steric hindrance. When the side chain group remained unchanged, the presence of 2-OH reduced the inhibitory activity of the corresponding Pd(II) complex of naphthylhydrazone to a certain extent. This result appears to be somewhat contrary to the anticancer activity of the metal complexes of 2-hydroxylphenylhydrazone mentioned above, suggesting the complexity of SAR studies. Therefore, the design of naphthylhydrazone without 2-OH and the synthesis and anticancer activity of its metal complexes should be further developed. It should be noted that the loss of 2-OH would also result in the loss of the *O*/*N*/*X*-tridentate coordination mode of naphthylhydrazone ligands. Therefore, special consideration should be given to the selection and design of the side chain groups so that the complexes can maintain the necessary coordination stability.

**Figure 30:**
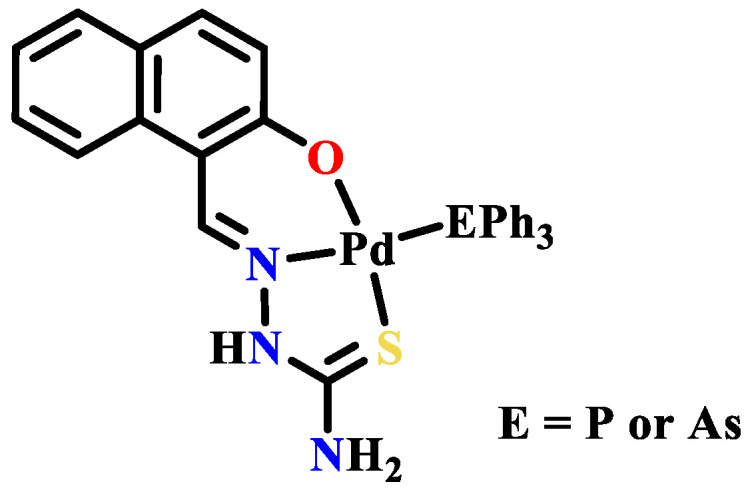
Two palladium(II) complexes of naphthylhydrazone with thiourea side chain with PPh_3_ and AsPh_3_ co-ligands, reported by R. Prabhakaran’s research group.

Meanwhile, R. Prabhakaran’s research group [105] also selected the same 2-hydroxylnaphthylhydrazone ligand with a thiosemicarbazone side chain and synthesized two similar naphthylhydrazone-Pd(II) complexes using PPh_3_ and AsPh_3_ as the co-ligands (Figure 30). The binding property of the two Pd(II) complexes with ct-DNA and BSA was also investigated. The results showed that the binding mechanism between the Pd(II) complex and BSA belonged to the static quenching. The BSA binding affinity of the Pd(II) complex with AsPh_3_ as a co-ligand was stronger. At the same time, compared with the previous work of Sarmistha Halder et al. [102], we further tested the in vitro activity towards two other cancer cell lines (A549 and HepG2), and the results showed that the two Pd(II) complexes still had certain inhibitory activity against the A549 and HepG2 cell lines, with IC_50_ values ranging from 9 μM to 22 μM, all of which were superior to the corresponding naphthylhydrazone ligands, and the sensitivity to HepG2 cells was slightly higher. In addition, the results showed that the Pd(II) complex could maintain its growth-inhibitory activity on typical cancer cells after the introduction of bulk co-ligands with steric hindrance, such as PPh_3_ and AsPh_3_.

**Figure 31:**
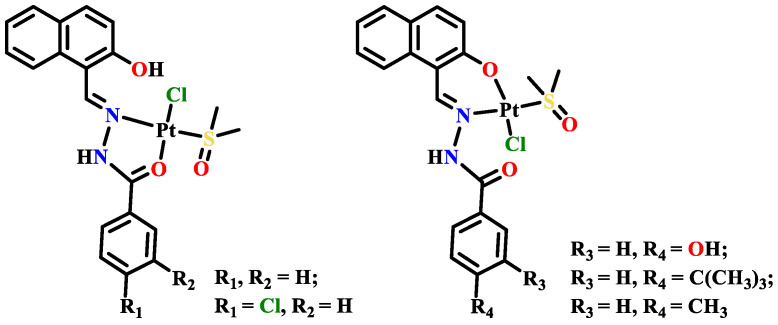
A series of Pt(II) complexes of naphthylhydrazone with different benzamide derivatives as side chains.

Recently, Feng Yang’s group [106] also reported five platinum(II) complexes of 2-hydroxynaphthylhydrazone with benzamide as a side chain, in which the benzene ring of the side chain was substituted with -Cl, -OH, and *tert*-butyl groups (Figure 31). These Pt(II) complexes were characterized by X-ray single-crystal diffraction analysis. The crystal structure of each Pt(II) complex showed that the naphthylhydrazone ligand was coordinated with Pt(II) in a *N*/*O*-bidentate mode, although there was 2-OH on the naphthalene ring, which could be due to the weak coordination affinity between the softer Pt(II) and the harder O atom. Therefore, the other two central Pt(II) coordination sites were occupied by a Cl atom and DMSO to complete the classical planar quadrilateral geometry of Pt(II). All five Pt(II) complexes were found to be cytotoxic compounds, and their antitumor activities were not lower than that of cisplatin, except for HeLa cells. The IC_50_ values ranged from 4.38 to 25.16 μM. By comparing the SAR, it was found that the anticancer activity of the complex was improved by the modification of the -OH and *tert*-butyl groups on the *para*-position of the benzene ring. Cell-uptake studies showed that platinum mainly existed in the membrane of the nucleus and mitochondria. In addition, all Pt(II) complexes could inhibit telomerase activity, target c-myc, and trigger apoptosis while also leading to cell cycle arrest in the S phase. In addition, Pt(II) complexes can also cause mitochondrial dysfunction, resulting in increased ROS production, decreased Δ*ψ_m_*, increased cytochrome C production, and an increased caspase-3/-9 ratio, showing the typical apoptotic characteristics of the mitochondrial pathway. These results strongly suggest that the Pt(II) complexes of naphthylhydrazone induce the apoptosis cancer cells via multiple mechanisms.

**Figure 32:**
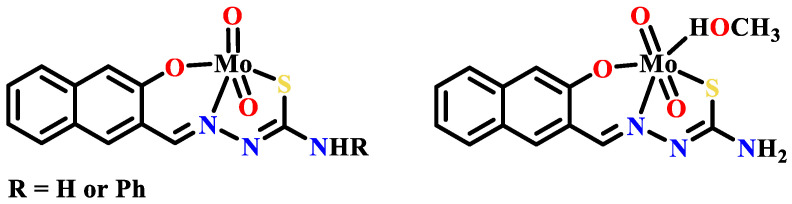
The Mo^VI^O_2_ complexes of naphthylhydrazone with thiourea or phenylthiourea side chains.

Early in 2010, Višnja Vrdoljak et al. [107] reported a series of +6 molybdenum(VI) complexes containing different substituent groups with aromatic hydrazones as ligands, including two Mo(VI) complexes with 2-hydroxylnaphthylhydrazone (Figure 32). This is also the first report detailing the biological activity of naphthylhydrazone-Mo(VI) complexes, in which the side chains of naphthylhydrazone were chosen to be thiourea and phenylthiourea, respectively. Among all of these complexes, the hydrazone ligands all coordinated with Mo(VI) in *O*/*N*/*S*-tridentate mode. The Mo(VI) complexes of two naphthylhydrazone showed significant differences in their anticancer activity. The Mo(VI) complex with a thiourea side chain showed no significant anticancer activity (IC_50_ > 5 μM) but was toxic to WI-38 normal cells. The Mo(VI) complex with a phenylthiourea side chain had the highest inhibitory activity against each cancer cell line (IC_50_ values were all <1 μM), even higher than the two positive control drugs, cisplatin and etoposide, indicating that it is a highly cytotoxic compound, but the inhibitory activity was still lower than that doxorubicin, a typical anthracycline anticancer drug. However, the author had also previously reported the anticancer activity of each hydrazone ligand mentioned above and found that the Mo(VI) complexes reported here showed activity that was no higher than the corresponding ligands, suggesting that Mo(VI) coordination could not further enhance the anticancer activity of these hydrazone compounds.

**Figure 33:**
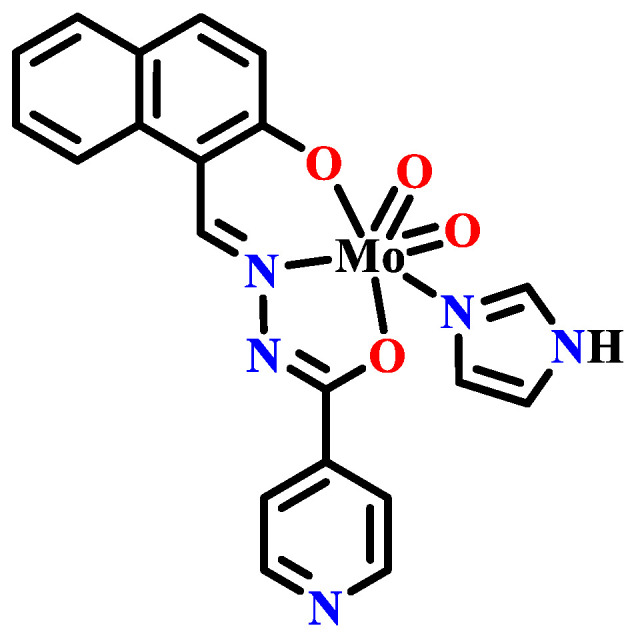
A Mo^VI^O_2_ complexes of naphthylhydrazone with isonicotinamide as the side chain and with imidazole as the co-ligand.

Very recently, Rupam Dinda’s group [108] also explored a new Mo(VI) complex of naphthylhydrazone with a +6-charged MoO_2_ moiety as the center (Figure 33). The side chain group of the 2-hydroxylnaphthylhydrazone was *p*-pyridinamide and coordinated with Mo(VI) in *O*/*N*/*O*-tridentate mode, while imidazole was used as the co-ligand to achieve a new six-coordinated Mo(VI) complex of naphthylhydrazone, which was compared with three other Mo(VI) complexes of phenylhydrazone. All four complexes were characterized by spectroscopic methods and X-ray single-crystal diffraction analyses. The results showed that the Mo(VI) complex of naphthylhydrazone could bind DNA more efficiently (3.57 × 10^4^ M^−1^) than the other three Mo(VI) complexes. Moreover, it also showed higher inhibitory activity against two cancer cell lines (HT-29 and HeLa), with IC_50_ values of 20.63 and 4.41 μM, respectively, which were also significantly higher than those of the other three Mo(VI) complexes of phenylhydrazone (IC_50_ = 60~177 μM). From the perspective of SAR, the anticancer activity of the Mo(VI) complex of naphthylhydrazone was higher than those of the other three, and its unique *p*-pyridinamide side chain group might also play a special role because other phenylhydrazone-Mo(VI) complexes have also used imidazole as a co-ligand. However, the differences in the side chain groups make their anticancer activity significantly weaker, suggesting that the imidazole co-ligand is not the key functional group contributing to anticancer activity.

**Figure 34:**
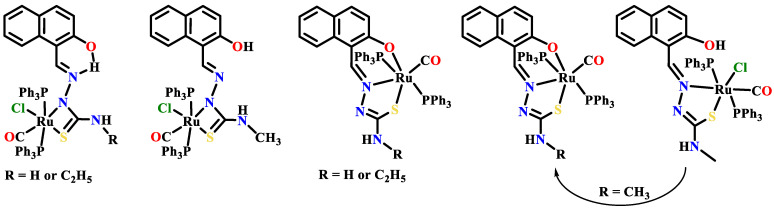
A series of Ru(II) complexes of naphthylhydrazone with thiourea/methylthiourea/ethylthiourea as the side chain, reported by R. Prabhakaran’s group.

As mentioned above, R. Prabhakaran’s group [109,110,111] not only reported on palladium(II) complexes of naphthylhydrazone, but also synthesized seven Ru(II) complexes via the coordination reaction of the lead compound, [RuHCl(CO)(PPh_3_)_3_], with the 2-hydroxylhydrazone ligand with a similar methyl/ethyl thiourea side chain. The central Ru(II) has a typical six-coordinated mode. In addition to the naphthylhydrazone ligand, there were also Cl, CO, and PPh_3_ co-ligands, as shown in Figure 34 below. When the R-group comprised H or two substituents, methyl/ethyl, the corresponding complexes could bind ct-DNA and BSA and inhibit the proliferation of tumor cells, showing good cytotoxicity. However, when the co-ligand was the same, regardless of whether the *N*/*S*-bidentate ligand or *O*/*N*/*S*-tridentate ligand was coordinated with Ru(II), when the side chain was ethyl thiourea, the inhibition activity of the complex towards A549 cell proliferation was significantly better than that of thiourea or methyl thiourea. In addition, the ethyl-substituted Ru(II) complex could be successfully embedded into mesoporous silica. The main components of the embedding complex began to release after 20 h, and the maximum level could be reached at 96 h [109].

Interestingly, we found that when the R-group was methyl, the *N*/*S*-bidentate chelation mode of the naphthylhydrazone ligand in the polar solvent gradually changed to the *O*/*N*/*S*-tridentate mode at room temperature; that is, the 2-OH of the naphthylhydrazone began to participate in the coordination. Unfortunately, the configuration transformation occurred in the solution. Therefore, it was difficult to detect the bioactivity of the converting intermediates. In terms of the anticancer activity, in the case of the same substituents, the *O*/*N*/*S*-tridentate complex had better inhibition activity than the *N*/*S*-bidentate complex against the A549 and HepG2 cancer cell lines. It was suggested that the increased electron delocalization in the five-membered ring and six-membered ring promoted the decrease in electron density on Ru(II), thus improving the penetration ability of the complex to cells. The density functional theory (DFT) study also showed that the Ru(II) complexes of *O*/*N*/*S*-tridentate mode had lower energy than the five-membered or four-membered ring Ru(II) complexes as well as a higher-tension *N*/*S*-bidentate mode [110].

**Figure 35:**
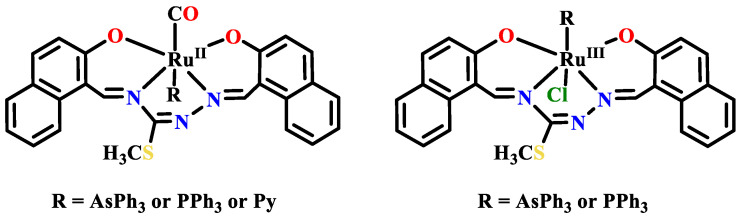
Ruthenium complexes of dinaphthylhydrazone ligands reported by R. Prabhakaran’s group (**left**) and Sellappan Selvamurugan’s group (**right**).

In 2013, Sellappan Selvamurugan et al. [112] also reported a new ruthenium(II) complex of 2-hydroxynaphthylhydrazone that had good symmetry and a side chain of 2′-hydroxynaphthyl *S*-methyl isopropyl thiosemicarbazide (called *S*-methylisothiosemicarbazone Schiff’s base by the authors) (Figure 35). They also synthesized three other similar phenylhydrazone-Ru(II) complexes. These Ru(II) complexes also had a typical octahedral geometry. The results showed that this Ru(II) complex of naphthylhydrazone ([Ru(CO)(PPh_3_)(L)]) had good scavenging activity against DPPH, ·OH, and ·NO, with IC_50_ values of 50.25, 6.17, and 13.96 μM, respectively. Compared with the ligands (67.87, 21.49, and 32.79 μM), the scavenging activity of the Ru(II) complexes was 1.3, 3.5, and 2.3 times higher. However, there was no significant difference in the antioxidant activity between it and the other three Ru(II) complexes. Furthermore, the inhibition of Ru(II) complexes against the human breast cancer cell line MCF-7 and the human skin cancer cell line A431 in vitro was determined by the 3-(4,5-dimethyl-2-thiazolyl)-2,5-diphenyltetrazoliumbromide (MTT) assay. The results were somewhat disappointing, as the activity of this Ru(II) complex was weaker than that of the other three Ru(II) complexes of phenylhydrazone. Subsequently, Govindan Prakash et al. [113] carried out similar work to Sellappan Selvamurugan. The ruthenium(III) complex was synthesized using the same *quasi*-symmetric 2-hydroxylnaphthylhydrazone ligand and was compared with the corresponding Ru(III) complexes of phenylhydrazone (Figure 35). The experiments determining the antioxidant activity showed that the two Ru(III) complexes had certain scavenging activities against DPPH, ·OH, ·NO, and H_2_O_2_ when using PPh_3_ and AsPh_3_ as co-ligands. The IC_50_ values of DPPH, ·OH, ·NO, and H_2_O_2_ of [RuCl(AsPh_3_)(L)] were 33.7, 19.2, 29.4, and 37.0 μM, respectively, and were 1~2 times of those of [RuCl(PPh_3_)(L)], indicating that the same type of co-ligand also affected the antioxidant activity of the complex. Compared with the Ru(II) complex mentioned above, the Ru(III) complexes studied in this paper only had 1/3~1/2 of the previous scavenging activity for ·OH and ·NO, but it significantly improved the scavenging activity for DPPH. Therefore, the different valence states of the metal center also affected the selectivity of the complexes when scavenging different free radicals. In addition, the complex [RuCl(AsPh_3_)(L)] also had certain inhibitory activity on the proliferation of MCF-7 cells, but the IC_50_ value was only 21.19 μM, which was significantly weaker than the corresponding phenylhydrazone-Ru(III) complexes.

**Figure 36:**
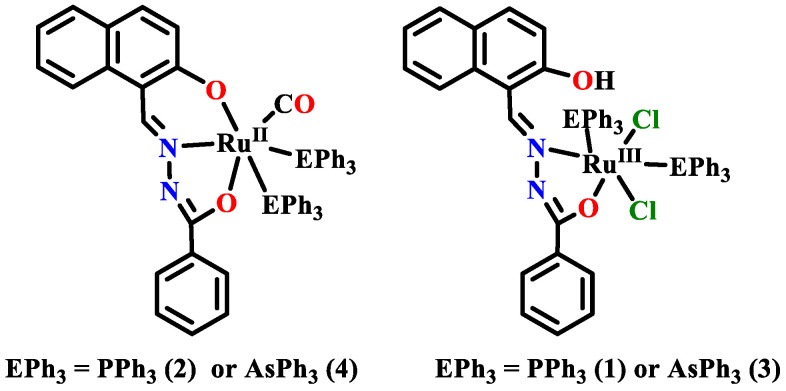
Ruthenium complexes of naphthylhydrazone with benzamide side chain reported by Eswaran Jayanthi’s group.

Eswaran Jayanthi et al. [114] reported Ru(III)/Ru(II) complexes of naphthylhydrazone with benzamide as a side chain (Figure 36). They found that regardless of whether or not KOH was added in the experiment, ruthenium complexes with different valence states formed, proposing a suitable mechanism for decomposition and alcohol dehydrogenation based on the Grubbs re-decomposition catalyst. The binding ability to DNA/HSA and the cytotoxicity of these four ruthenium complexes with different co-ligands (Cl, CO, and As/PPh_3_) can be listed in the order of **1** > **3** > **2** > **4**. These ruthenium complexes all promoted the release of lactate dehydrogenase (LDH), NO, and ROS in A549 cells, suggesting that the complexes could induce apoptosis and inhibit the proliferation of cancer cells. The combination of the biological prospects associated with +2- and +3-charged ruthenium complexes really revealed the influence of the structure and oxidation state of the metallodrugs on their biological activities. When 2-OH was not deprotonated, the naphthylhydrazone ligand coordinated with Ru(III) through the *N*/*O*-bidentate chelation mode, and the retained 2-OH provided an effective site for hydrogen bonding with biomolecules. If 2-OH was deprotonated, then phenole-O participated in the coordination, while the same naphthylhydrazone ligand coordinated with Ru in an *O*/*N*/*O*-tridentate chelation mode, where ruthenium was +2-charged. Additionally, the Ru(III) complexes with PPh_3_ as the co-ligand showed higher activity than those with AsPh_3_ as the co-ligand.

### 3.2. Anticancer Metal Complexes of Anthrahydrazone

In terms of structure, anthracene has only one more aromatic ring than naphthalene, but it is undeniable that since the discovery of anthracycline (doxorubicin, daunorubicin, etc.) [115] in the 1960s, more and more compounds based on the anthracene structure have shown effective and extensive inhibitory effects on malignant tumors. Until now, anthracyclines remain one of the most commonly used drugs in chemotherapy regimens for various malignancies. However, reports on the metal complexes of anthrahydrazone that are based on the parent structure of anthracene are not as common as those of naphthylhydrazone. However, as a less-covered field, scholars around the world are still carrying out more and newer research work in this field.

#### 3.2.1. The First Transition Metal Complexes of Anthrahydrazone with Anticancer Activities

Since an aldehyde group is necessary for the synthesis of hydrazone and since the 9-anthracene aldehyde is the most common and easily available anthracene aldehyde, metal complexes of anthrahydrazone mainly refer to those of 9-anthrahydrazone, as shown in the following section. These reports are mostly from the past 10 years, indicating that the research work in this area still has broad enough space to expand and deepen.

**Figure 37:**
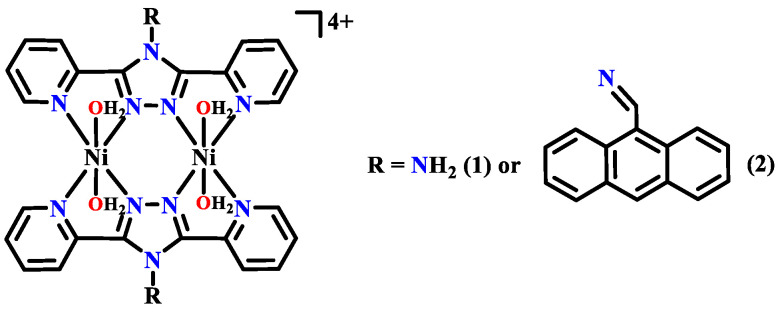
Symmetric binuclear nickel(II) complexes of 9-anthrahydrazone with triazole derivative as a side chain.

A special 9-anthrahydrazone ligand was reported in 2013 by Sengottaiyan Poornima et al. [116]. They synthesized a novel 9-anthrahydrazone with a triazole derivative as a side chain via the condensation of a 9-anthracene aldehyde with 3,5-bis(2′-pyridine)-1,2,4-triazole, as shown in Figure 37 below. In particular, in the nickel(II) complex synthesized from this anthrahydrazone ligand, the hydrazone bond does not participate in coordination, but chelates two Ni(II) atoms with two pyridin-N atoms and 1,2-N atoms of triazole to form a highly symmetrical binuclear nickel(II) complex. Since the whole anthrahydrazone is neutral, the coordination unit was +4-charged, making the whole complex a cationic water-soluble nickel(II) complex (**2**). A simple hydrazone ligand with a triazole side chain and nickel(II) complex (**1**) was also synthesized for comparison. The authors studied their binding abilities to DNA and BSA and their anticancer activity. The results show that the two Ni(II) complexes interact with DNA in intercalative mode, and the binding constants were 2.36 × 10^5^ and 4.87 × 10^5^ M^−1^, respectively. The binding constants of the complex with BSA were 0.71 × 10^6^ and 5.62 × 10^6^ M^−1^, respectively, suggesting that the complex could induce changes in protein conformation. The calculated results of it binding with DNA and BSA also showed that the presence of an anthracene group increased the area of the conjugated plane and the hydrophobicity of the anthracene ligand, which was conducive to its binding affinity to the two biological macromolecules. In addition, the Ni(II) complex **2** of triazolanthrahydrazone also showed twice the inhibitory activity of Ni(II) complex **1** without anthracene on HeLa and BeWo cells, with IC_50_ values of 13.30 and 17.06 μM, respectively, suggesting that the presence of anthracene could increase the proliferation inhibition of the cancer cells.

**Figure 38:**
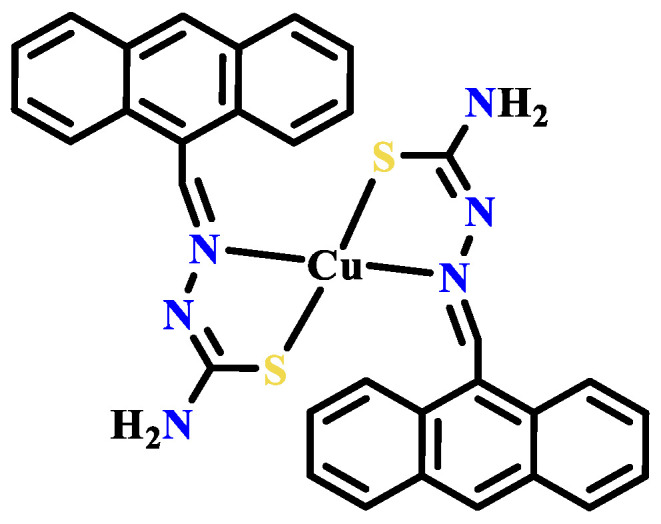
A copper(II) complex of 9-anthrachydrazone with thiourea as a side chain.

Subsequently, Anup N. Kate et al. [117] synthesized a copper(II) complex of 9-anthrachydrazone in *N*/*S*-bidentate mode using thiourea as a side chain (Figure 38) and characterized its coordination structure by means of spectroscopy, electrochemistry, and crystallographic methods. The results showed that the anthrahydrazone ligand and its Cu(II) complex could effectively bind DNA and BSA through intercalation and hydrophobic interaction, respectively. In contrast, the Cu(II) complex had a dynamic quenching mode on the fluorescence of BSA, while the anthrahydrazone ligand had a static quenching mode. Even without the addition of other reagents, the Cu(II) complex could effectively cleave pBR322 plasmid DNA under the irradiation of 365 nm of UV light. Due to the inherent fluorescence characteristics of the anthracene ring, the anthrahydrazone ligand and its Cu(II) complex were found to mainly be distributed in the nucleus of HeLa cells under a fluorescence microscope, which further suggests that the nucleus and DNA might be its potential anticancer targets. Meanwhile, the Cu(II) complex induced apoptosis in HeLa cell more strongly than the ligand.

**Figure 39:**
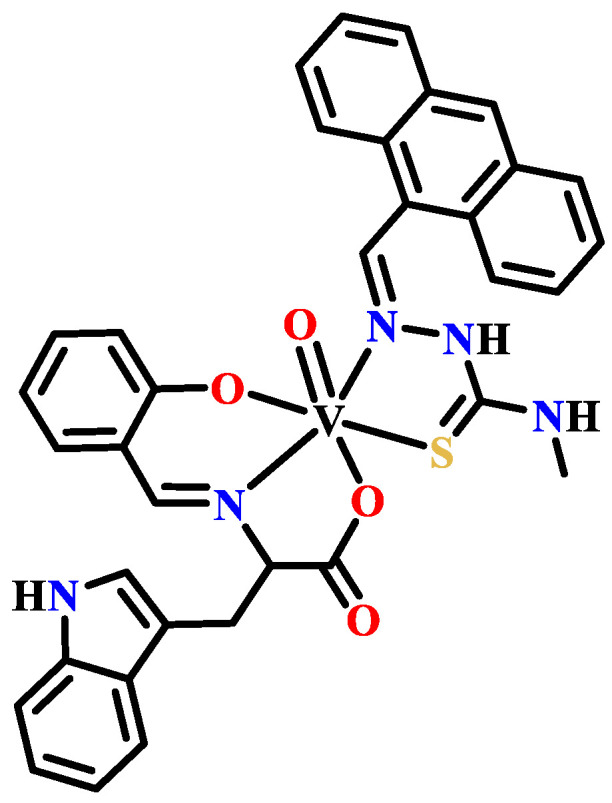
A vanadium(IV) complex of 9-anthrahydrazone with methylthiourea as a side chain.

Alvin A. Holder’s team [118,119] also attempted to synthesize a novel vanadium(IV) complex and cobalt(III) complex of 9-anthrahydrazone with methyl thiourea as a side chain by introducing different co-ligands (Figure 39). In 2012, they reported the synthesis of a lead complex with tryptophan, salicylic acid, and VOSO_4_ and further mix-coordinated with the above 9-anthrahydrazone ligand to synthesize a new V(IV) complex as well as two other V(IV) complexes of non-anthrahydrazone ligands. Interestingly, when dissolved in DMSO solution, these V(IV) complexes were found to be oxidized to +5-charged V(V) complexes in air, as characterized and confirmed by ESI-MS and ^51^V-NMR. Their in vitro cytotoxicity towards three typical colon cancer cell lines and the normal colonic myofibroblasts CCD-18Co was tested. The results showed that the proliferation of various colon cells was increasingly inhibited according to the incubation time of the complex with the cells, but the inhibitory activities of the anthrahydrazone-V(IV) complex on cancerous and non-cancerous colon cells were different, and the IC_50_ value of the complex was 208.0 μM after 72 h treatment with CCD-18Co cells. The IC_50_ values of HT-29, HCT-116, and Caco-2 were 100.3, 115.0 and 147.1 μM, respectively, under the same treatment time, indicating a certain toxicity selectivity to cancer cells. However, according to the IC_50_ value, the anticancer activity of this special V(V)/V(IV) complex was relatively weak, and its inhibitory effect on colon cancer cells was much lower than that of etoposide. However, its toxicity towards normal colon cells was also much lower than that of etoposide, so it still showed a certain medicinal prospect [118].

**Figure 40:**
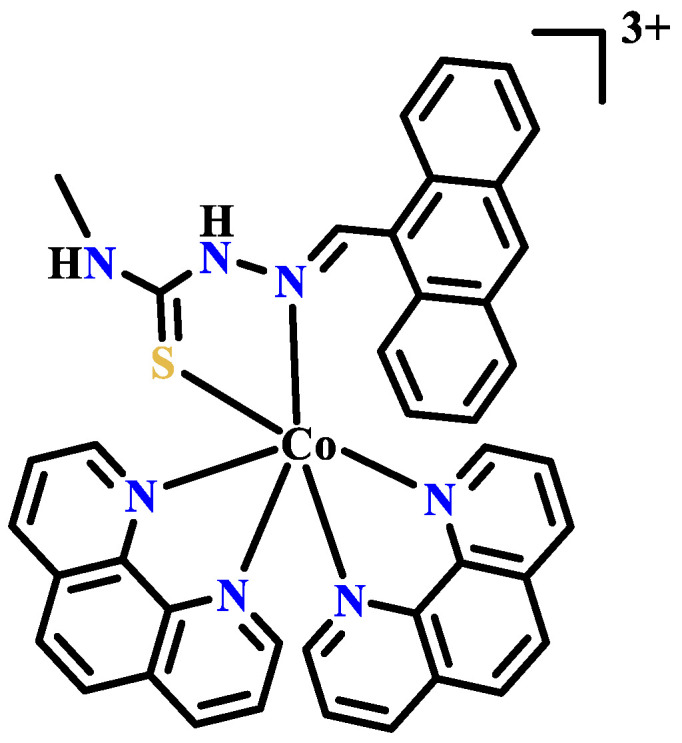
A cobalt(III) complex using the same 9-anthrahydrazone ligand by introducing *o*-phenanthroline as a co-ligand.

Subsequently, in 2020, they further synthesized a novel Co(III) complex using the same 9-anthrahydrazone ligand by introducing *o*-phenanthroline as a co-ligand (Figure 40) [119]. The 9-anthrahydrazone and two *o*-phenanthrenes both coordinated with Co(III) via *N*/*S*- and *N*/*N*-bidentate chelation modes, respectively, to form a hexahedral complex. Since all three ligands are electrically neutral, this coordination unit existed as a +3-charged cationic species. Similar to most anthrahydrazone complexes, this complex could interact with ct-DNA in the intercalative mode, but the binding intensity was weak, with a binding constant (*K*_b_) of 1.6 × 10^4^ M^−1^, which is much lower than that of the 9-anthrahydrazone ligand (*K*_b_ = 8.1 × 10^5^ M^−1^). This may be related to the steric hindrance of this octahedral Co(III) complex. On the other hand, the anthrahydrazone ligand and its Co(III) complex showed good inhibition ability on Topo I and IIα, suggesting that topoisomerase is still a potential anticancer target of these anthracycline derivatives. At the cellular level, the ligand did not significantly inhibit cell proliferation, but the IC_50_ value of the Co(III) complex was 34.4 ± 5.2 μM after being treated with 4T1-Luc cells for 24 h, and it could activate the caspase 3/7 cascade through the mitochondrial pathway, ultimately inducing cell apoptosis. These results indicated that as a metal center, Co(III) played an important role in the anticancer activity of this anthrahydrazone complex.

**Figure 41:**
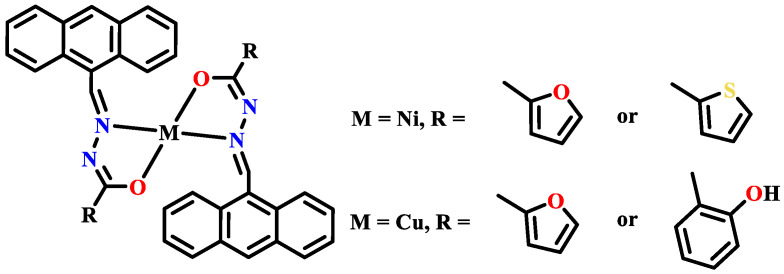
Co(II)/Ni(II) complexes of 9-anthrahydrazone with different heterocyclic acylamides as a side chain.

Neethu K.S et al. [120] introduced five- and six-membered heterocycles (such as furan, thiophene, and phenol) into the amide side chain of 9-anthrahydrazone, synthesized a series of new 9-anthrahydrazone ligands, and then coordinated with nickel(II) and copper(II) to synthesize four respective complexes of anthrahydrazone (Figure 41). The four complexes all formed to have a planar quadrilateral coordination geometry, and the two anthrahydrazone ligands symmetrically coordinated to the central Ni(II)/Cu(II) via *N*/*O*-bidentate chelation. The interaction of the complexes with DNA and BSA was studied by UV—Vis and fluorescence spectral analyses and viscosity experiments. The results showed that the complex also interacted with DNA, and the binding constants were in the range of 5.453 × 10^3^~7.143 × 10^5^ M^−1^. Meanwhile, the complex also showed a strong binding effect with BSA. The MTT assay was used to test the inhibitory activity of each complex against the colon cancer cell line (HCT-15). The corresponding IC_50_ values ranged from 13.90 μM to 18.26 μM, which was even stronger than cisplatin (IC_50_ = 25.4 μM). In addition, the cytotoxicity of each complex to the normal skin cell line (L929) was very low, with IC_50_ > 150 μM, similar to cisplatin. These results indicated that the complexes had some cytotoxic selectivity against colon cancer cells. Further studies showed that all of the complexes could induce the apoptosis of cancer cells and had good scavenging ability on DPPH-free radicals.

**Figure 42:**
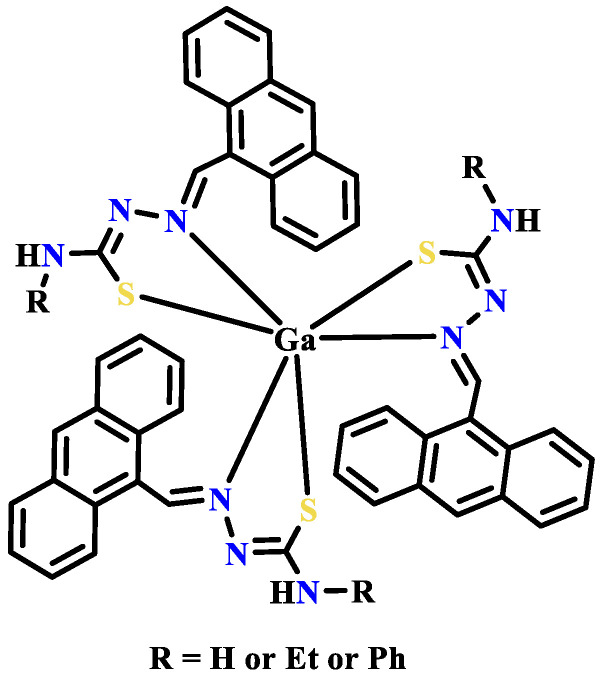
Three gallium(III) complexes of anthrahydrazone with (*N*-ethyl/phenyl)thiourea as a side chain.

In recent years, Floyd A. Beckford’s group has carried out a number of valuable studies on the metal complexes of 9-anthrahydrazone. Gallium(III) complexes of anthrahydrazone with (*N*-ethyl/phenyl) thiourea as a side chain were reported for the first time (Figure 42) [121]. In each complex, three anthrahydrazone ligands coordinated with Ga(III) via *N*/*S*-bidentate mode, where each deprotonated thiol group gave a −1 valence, making the whole complex electrically neutral. The Ga(III) complexes with different thiourea side chains showed significant anticancer activity against two human colon cancer cell lines, HCT-116 and Caco-2, that increased in a time-dependent manner within 24~72 h, with IC_50_ values ranging from 4.7 to 44.1 μM. However, their cytotoxicity against the normal colon cell line CCD-18Co was much lower. Through SAR comparison, the Ga(III) complex of the thiourea side chains showed the highest activity, with an IC_50_ of 4.7 μM after 72 h of treatment, while the complex of the *N*-phenylthiourea side chain showed the lowest activity, with an IC_50_ of 44.1 μM for Caco-2 cells, which was only 1/10 of that of the Ga(III) complex with the thiourea side chain. This means that steric hindrance of side chain groups is not conducive to the anticancer activity of this variety of complexes. In addition, the three complexes could intercalate with the DNA and cleaved DNA with binding constants, *K*_b_, with values ranging from 7.46 × 10^4^ to 3.25 × 10^5^ M^−1^. Compared with the DNA-binding analysis, there seemed to be no direct correlation between the DNA-binding ability of the complex and its in vitro anticancer activity. According to the calculated binding constants between has and the complexes (in the range of 10^4^~10^5^ M^−1^), the higher the steric hindrance of the side chain substituents, the lower the binding ability. They subsequently calculated the binding property of the complex with DNA, ribonucleotide reductase, and HSA via molecular docking. However, the results showed that the complex with the best anticancer activity also had the largest binding constant with DNA.

Our research group at Guangxi Normal University, Guilin, China also started to study the metal complexes of 9-anthrahydrazone derivatives about 10 years ago. Our work was inspired by the anthrahydrazone structure of bisantrene, which had been developed and marketed for clinical use in cancer chemotherapy but has not achieved further success due to its poor water solubility and high toxicity. Bisantrene has a symmetric anthracene dihydrazone structure and has shown high cytotoxicity via the introduction of an imidazoline group with strong alkalinity on both side chains of anthracene. Therefore, we used sisantrene as the parent structure to improve the anticancer effect of this anthrachydrazone through structural modification and the formation of metal complexes.

**Figure 43:**
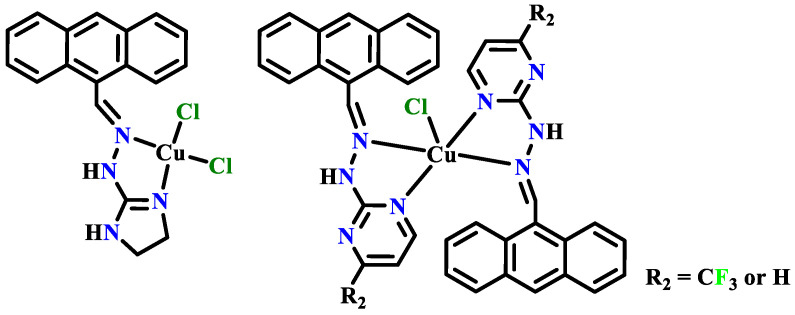
Copper(II) complexes of 9-anthrahydrazone with imidazoline or pyrimidine as the side chain.

At first, we chose to retain one side chain of bisantrene to achieve 9-anthrahydrazone with imidazolinyl as side chain (9-imidazolinyl anthrahydrazone or anthracene-9-imidazolinylhydrazone, 9-AIH). A series of platinum(II) complexes of 9-AIH were thus synthesized, and their anticancer activities were tested in vitro. However, the results showed that the activities of these 9-AIH-Pt(II) complexes, represented by the cisplatin-like type, were all lower than those of the 9-AIH ligand, although their toxicities to HL-7702 normal liver cells were also lower [122]. In 2015, we discovered and reported a highly active copper(II) complex of 9-AIH (Figure 43) [123]. This anthrahydrazone ligand coordinated with Cu(II) via *N*,*N*-bidentate chelation and formed a planar quadrilateral configuration together with two Cl atoms. The Cu(II) complex showed significant anticancer activity in vitro, with IC_50_ values ranging from 0.94 to 3.68 μM against five typical human cancer cell lines and were significantly higher than those of 9-AIH (4.86 to 12.13 μM). This Cu(II) complex could cause cancer cell death by blocking the cell cycle and activating the ROS-related mitochondrial pathway. Under the same conditions, the ligand did not exhibit such an action mechanism, fully demonstrating the unique anticancer mechanism of Cu(II), which gave us a good reference value.

Subsequently, we attempted to change the imidazoline side-chain group of bisantrene into other *N*-containing aromatic rings to obtain more new metal complexes of anthrahydrazone so that more comprehensive SAR studies could be carried out. Two new copper(II) complexes were thus synthesized by replacing imidazoline with pyrimidine on the side chain of anthrahydrazone to obtain pyrimidine anthrahydrazone (PMAH), which also contains two N atoms on the ring (Figure 43) [124]. In these two complexes, the PMAH ligand coordinated with Cu(II) through azomethine-N and pyrimidin-N via a *N*,*N*-bidentate chelation mode. The two PMAHs and a Cl atom formed a five-coordination tetragonal cone configuration. The only difference between the two complexes was whether there was a -CF_3_ group on the pyrimidine ring or not. Both Cu(II) complexes could bind to DNA by intercalation and were also effective Topo I inhibitors. They also showed significant proliferation inhibition on the six cancer cell lines tested. Through SAR comparison, it was found that the presence of the electron-withdrawing group -CF_3_ on the pyrimidine obviously weakened its activity and was speculated to be still related to steric hindrance. However, the Cu(II) complex of higher-activity PMAH without a substituent could still maintain a stable coordination state in H_2_O in the presence of HSA. Additionally, it could also block the cell cycle and induce cell apoptosis. We further found that this Cu(II) complex showed a significant in vivo inhibitory effect on T-24 tumor proliferation in a xenograft tumor model of nude mice. Although the in vivo inhibitory effect of the Cu(II) complex was still weaker than the clinical drug, cisplatin, this was the first in vivo pharmacological study on the metal complex of anthrahydrazone.

**Figure 44:**
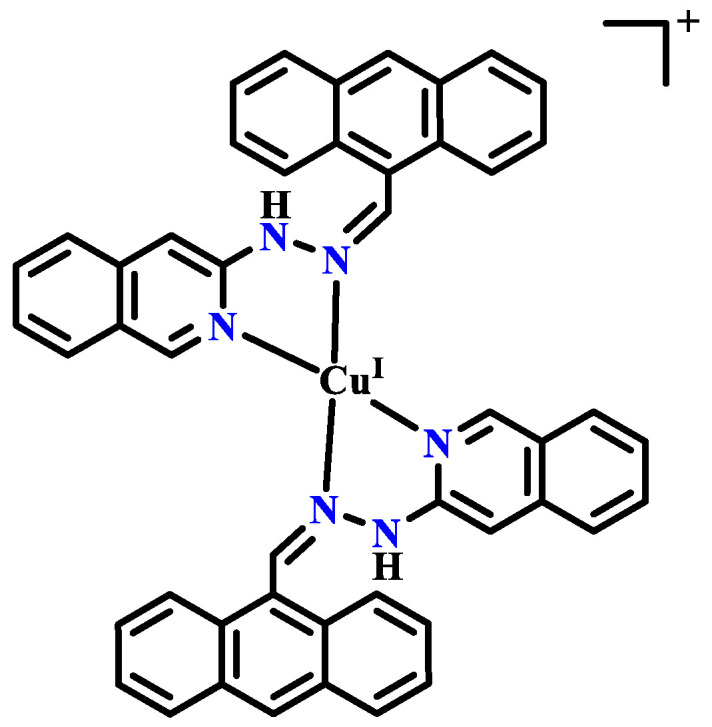
The first copper(I) complex of 9-quinolinylhydrazone showing significant and broad-spectrum anticancer activity in vitro.

On the other hand, we continued to adjust the side chain of 9-anthrahydrazone and introduced another pharmacodynamic group, quinoline, to obtain a new anthrahydrazone, anthracene-9-quinolinylhydrazone (AQH), which was used as a ligand to synthesize and characterize four new transition metal complexes (Cu(I), Co(II), Ni(II), and Zn(II)) (Figure 44) [125]. Interestingly, the redox reaction occurred when the AQH ligand was chelated with Cu(II), thus accidentally obtaining the first Cu(I) complex of anthrachydrazone. More surprisingly, only the Cu(I) complex of this series of AQH—metal complexes showed significant and broad-spectrum anticancer activity in vitro. Although the activity of the Cu(I) complex of AQH was slightly weaker than those of the above-mentioned Cu(II) complexes of 9-AIH and 9-PMAH, this further suggested that in addition to different types of the central metals, the changes in the redox state also had a significant effect on anticancer activity. The anticancer mechanism of the AQH-Cu(I) complex against the most sensitive MGC-803 cells suggests that it can significantly induce the apoptosis of cancer cells through the mitochondrial pathway rather than by blocking the cell cycle. Further EPR and electrophoresis results showed that the presence of Cu(I) could catalyze the generation of ·OH and other radicals in the H_2_O_2_ system. Although no significant Cu(II) signals were detected at the cellular level, the total ROS level in MGC-803 cells was weakened after incubation with AQH-Cu(I). Considering the complexity of the cell and other possible reductants, we still could not exclude that the potential redox activity between Cu(I) and Cu(II), which plays a key role in the anticancer activity of the complex. Meanwhile, autophagy was also observed in cancer cells treated with AQH-Cu(I), suggesting the complexity of its anticancer mechanism. These results suggested that Cu(I) induced multiple anticancer mechanisms and played an important role in the anticancer activity of the complex. This study also further enriched the structural library of metal complexes of anthrahydrazone, as well as the related SAR research.

In recent years, based on studies of the metal complexes of 9-anthrahydrazone, our research group started to carry out non-symmetric synthesis on the two side chains based on the structure of 9,10-anthracene dialdehyde by retaining the -CHO group on one side of anthracene and forming various hydrazones containing *N*-heterocyclic side chains (such as pyrimidine, imidazoline, benzothiazole, etc.) on the other side, which we named 9-aldehyde-10-anthrahydrazone [31,32,33,126]. A series of novel aldehyde anthrahydrazone ligands and their metal complexes were thus synthesized and explored. To our knowledge, this is the first time that a non-symmetric anthrahydrazone with different groups on two sides of the anthracene ring has been synthesized. It should be also acknowledged that such research was derived from bisantrene, which has a symmetrical anthracene bishydrazone structure. Additionally, the aim of retaining the aldehyde group on the other side of the anthrahydrazone is for the condensation of different amino compounds via the aldehyde group, thus creating a large number of non-symmetric anthrahydrazone derivatives, in order to provide a wealth of new compounds for the further exploration of their biological activities.

**Figure 45:**
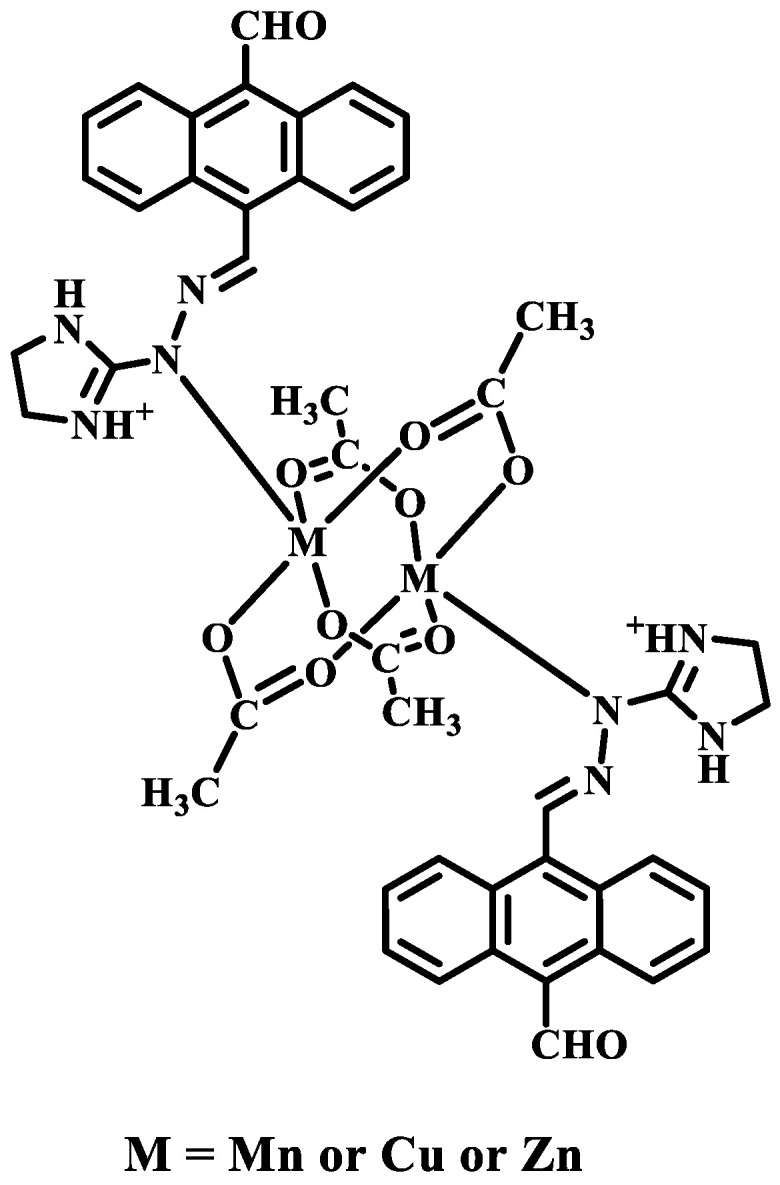
Binuclear transition metal complexes of *p*-aldehyde anthrahydrazone with the same imidazoline side chain as bisantrene with bridging coordinated by four acetate co-ligands.

The transition metal complexes of 9-aldehyde-10-anthrahydrazone that we obtained mainly contained those of Mn(II), Cu(II), and Zn(II) (Figure 45). Most of the complexes of *p*-aldehyde anthrahydrazone had a binuclear structure. The results showed that Cu(II) complexes of *p*-aldehyde anthrahydrazone had higher anticancer activity in vitro. In the Cu(II) complexes, the side chain groups of anthrahydrazone also had a significant effect on the activity, and the activity sequence was benzothiazole > imidazoline > dimethylpyrimidine. Among the three binuclear metal complexes of *p*-aldehyde anthrahydrazone with the same imidazoline side chain as bisantrene, the order of activity was Mn > Zn > Cu. Therefore, it is difficult to accurately summarize the SAR from the existing limited metal complexes of *p*-aldehyde anthrahydrazone. Additionally, the metal center and side chain groups as well as the substituent seem to have the most critical influence on the anticancer activity of the complexes. However, factors such as the solubility and coordination mode of the complex should not be ignored. Therefore, more metal complexes of *p*-aldehyde anthrachydrazone need to be synthesized and explored to build a richer library of this kind of metal complexes for SAR studies.

#### 3.2.2. The Second and Third Transition Metal Complexes of Anthrahydrazone with Anticancer Activities

**Figure 46:**
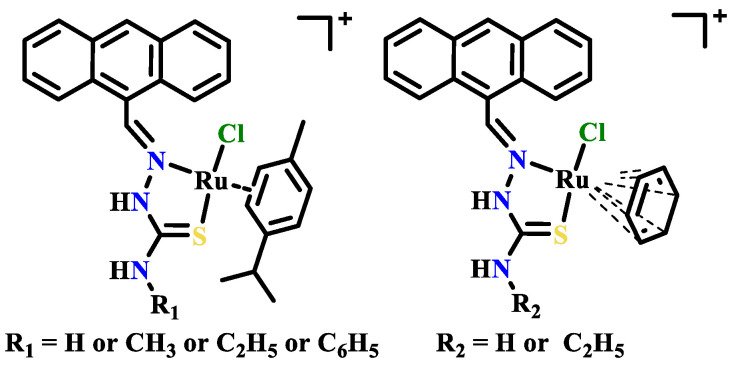
Organo-Ru(II) complexes of 9-anthrachydrazone with *N*-(methyl/ethyl/phenyl)-substituted thiourea as side chains, as reported by Floyd A. Beckford’s group.

As early as 2009, Floyd A. Beckford et al. [70,127,128] designed and synthesized a series of 9-anthrachydrazones with *N*-substituted thiourea as side chains and used them as the active ligands to coordinate with Ru(II) through *N*/*S*-bidentate chelation. Benzene and *p*-cymene were used as Π-bonding co-ligands. Four corresponding organometallic Ru(II) complexes were synthesized and characterized, and their structures are shown in Figure 46. The anticancer activities of these Ru(II) complexes against several human cancer cell lines were compared via an in vitro test. The results showed that the *N*-substituent group had an obvious effect on the anticancer activity of Ru(II) complexes: CH_3_ > H > C_6_H_5_ > C_2_H_5_ [70,127]. In addition to the metal complex with a *N*-ethyl thiourea side chain, the MDA-MB-231 cell line was the most sensitive to the other three Ru(II) complexes (IC_50_ = 2~9 μM), while cisplatin only had inhibitory activity of 730 μM. Thus, the Ru(II) complexes of anthrahydrazone retained the high cytotoxic selectivity of anthracyclines to breast cancer cells. The *N*-ethyl-substituted complex showed weak and unsatisfactory inhibitory activity on colon cancer cells. After 72 h, the IC_50_ values of two kinds of colon cancer cells, HCT-116 and Caco-2, were only 200~225 μM, and there were no significant differences in their inhibitory effect on normal colon tissue cells. However, the complex showed a strong binding effect with HSA and DNA and could significantly inhibit the activity of Topo II, suggesting that it might be able to inhibit other different types of cancer cells. Then, an organometallic Ru(II) complex with benzene as the Π-bonding co-ligand was synthesized by a novel microwave-assisted synthesis method. The side chain group of anthrahydrazone was thiourea or *N*-ethyl thiourea [128]. In previous studies, Ru(II) complexes with a *N*-ethyl thiourea side chain had the lowest activity, but the authors continued to select the Ru(II) complex replaced by *N*-ethyl, which may be related to its strong binding affinity with HSA/DNA and Topo II inhibition. The results showed that the aryl Ru(II) complexes of the two anthrahydrazones could bind with DNA and HSA intensively and inhibit the proliferation of the two colon cancer cells, HCT-116 and cacO-2. The activity of the *N*-ethyl-substituted Ru(II) complex was 1~2 times that of the non-substituted Ru(II) complex. Furthermore, the anticancer activity of the Ru(II) complex against both cancer cells was also higher than that of the Ru(II) complex with the same side chain but with the co-ligand *p*-cymene, suggesting that the corresponding Ru(II) complex might have higher in vitro anticancer activity when there are no substituents on the benzene ring of the co-ligand [70,127,128].

**Figure 47:**
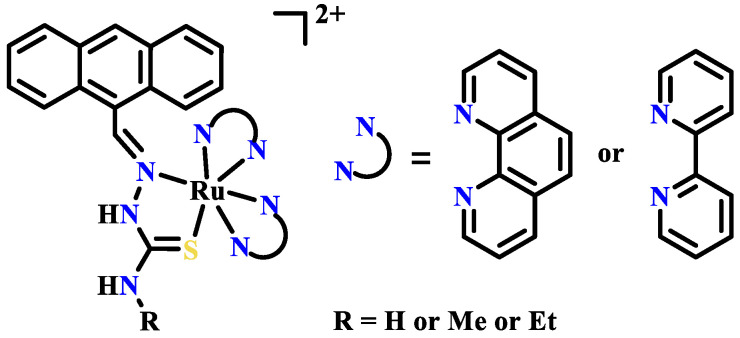
Ru(II) complexes of 9-anthrachydrazone with *o*-phenanthrene or bipyridine as the co-ligand.

In addition, Floyd A. Beckford’s group continued to introduce different co-ligands, such as *o*-phenanthrene and bipyridine, and also synthesized and characterized two new series of mixed ligand Ru(II) complexes of 9-anthrahydrazone using a microwave-assisted method. Thiourea, methyl thiourea, and ethyl thiourea were selected to be the side chains of each series of anthrahydrazones (Figure 47) [129]. The coordination configurations of these Ru(II) complexes are similar to those of the mixed ligand Co(III) complexes of 9-anthrahydrazone and *o*-phenanthroline/bipyridine, as previously reported by Alvin A. Holder et al. [119]. Competitive fluorescence analysis of DNA and DNA viscosity experiments conducted using ethidium bromide (EB) as a probe showed that the two Ru(II) complexes bind to DNA via intercalation with a strong binding ability. The six Ru(II) complexes significantly inhibited the proliferation of two types of colon cancer cells (HCT-116 and HT-29) and two types of breast cancer cells (MDA-MB-231 and MCF-7). The inhibitory activity on breast cancer cells was much higher than that of cisplatin. Through SAR comparison, the anticancer activity of the Ru(II) complexes with *o*-phenanthroline as a co-ligand was significantly higher than those with bipyridine as a co-lignad. On the other hand, with the substitution of a methyl or ethyl group to the *N*- of thiourea side chain, the anticancer activity also increased. This further indicates that the co-ligand and the side chain substituents of anthrahydrazone were important factors affecting the anticancer activity of the complex under the same coordination mode. In addition, anthrahydrazone itself also has a certain level of cytotoxicity, and the coordination with metal ions could exert a positive synergistic effect, significantly enhancing and regulating the anticancer activity and action mechanism of the complex.

**Figure 48:**
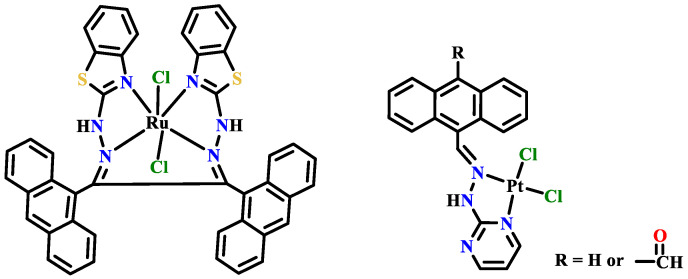
The Ru(II) and Pt(II) complexes of 9-anthrachydrazone with benzothiazole and pyrimidine as side chains.

Recently, we also synthesized a 9-anthrahydrazone ligand and its Ru(II) complex using benzothiazole as a side chain (Figure 48) [130]. It was found that the Ru(II) complex showed better anticancer activity than the ligand and showed obvious cell selectivity. In addition, we attempted to compare the anthrahydrazone with the aforementioned non-symmetric aldehyde anthrahydrazone via SAR and thus synthesized two Pt(II) complexes of anthrahydrazone with pyrimidine side chains: PMAH-Pt and APMAH-Pt (Figure 48) [131]. Among the two complexes, the anthrahydrazone ligand coordinated with Pt(II) in *N*/*N*-bidentate chelation mode via azomethine-N and pyrimidine-N together with two Cl atoms to form a typical planar quadrilateral configuration similar to that of cisplatin. The only difference between the two Pt(II) complexes is whether there is 10-CHO on the other side of the anthrahydrazone. The results of in vitro antitumor activity screening showed that the presence of 10-CHO in the anthrahydrazone was not conducive to increasing the overall cytotoxicity, which was possibly due to the enhanced steric hindrance or polarity of the anthracene ring since DNA and topoisomerase are regarded as the anticancer targets of anthracyclines. The anticancer mechanism of PMAH-Pt, which had higher activity, was found to be similar to that of PMAH-Cu with the same ligand. At the cellular level, it could arrest the cell cycle of T-24 and MGC-803 cells in G2/M phase and induce cell apoptosis. At the molecular level, it could bind to DNA by means of intercalation and effectively inhibit the activity of Topo I, making it an effective Topo I inhibitor. It should be noted again that although the presence of a *p*-aldehyde group might reduce the cytotoxicity of anthrahydrazone and its metal complexes to a certain extent, it also reduces the toxicity of such anthrahydrazone derivatives to normal cells, thus providing a basis for its transformation from highly cytotoxic compounds to functional anticancer compounds. However, the retention of 10-CHO provides a pre-embedded site for introducing functional groups and also provides more possibilities for the design of metal complexes of non-symmetric anthrahydrazones.

**Figure 49:**
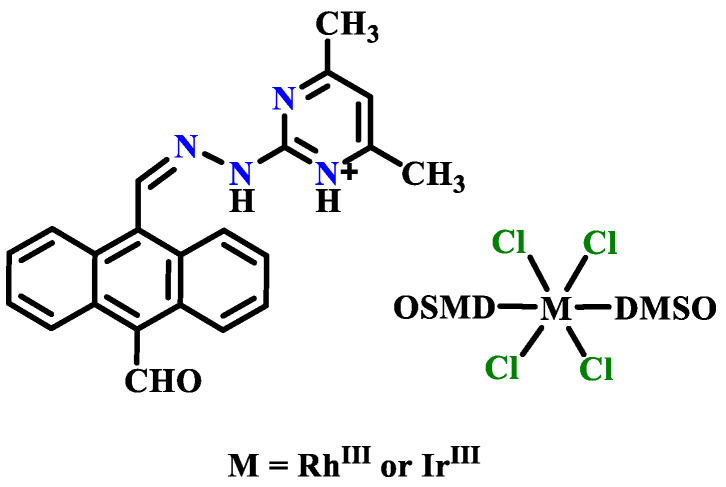
Two special ionic Rh(III)/Ir(III) complexes of *p*-aldehyde anthrachydrazone with a dimethylpyrimidine side chain.

In addition, we also reported two other ionic-type metal complexes of aldehyde anthrachydrazone with 4′/6′-dimethylpyrimidine side chains, AMPMAH-Rh/Ir, which were different from the above-mentioned ones because of the substitution of 4′- and 6′-CH_3_ on the pyrimidine ring, as shown in Figure 49 [33]. The characteristics of the two complexes were that one N atom of the pyrimidine of anthrahydrazone was protonated to present a +1 valence, and the central Rh(III) or Ir(III) existed in the form of anionic [Rh/IrCl_4_(DMSO)_2_]^−^, presenting a −1 valence and thus keeping the whole complex electrically neutral. Unfortunately, their anticancer activities were not significant in vitro, except those of T-24 and NCI-H460, but they were not toxic to the normal human liver cell line HL-7702, indicating that their hepatotoxicity was very low and significantly lower than cisplatin. AMPMAH-Ir in particular could effectively inhibit the proliferation of two typical human cancer cell lines, T-24 and NCI-H460, with IC_50_ values of 9.05 μM and 10.98 μM, respectively, and had no significant toxicity to normal liver cells (IC_50_ > 20 μM), showing good cytotoxic selectivity. According to existing research results, although the ionic complexes did not directly coordinate with the metal ions on the surface, the species that existed in solution were complicated and involved some coordinated ligand–metal species that are likely to have key proliferation-inhibitory activities. This is one of the reasons why ionic complexes are also significantly more active than ligands [132].

### 3.3. Anticancer Metal Complexes of the Other Polycyclic Aromatic Hydrazones

**Figure 50:**
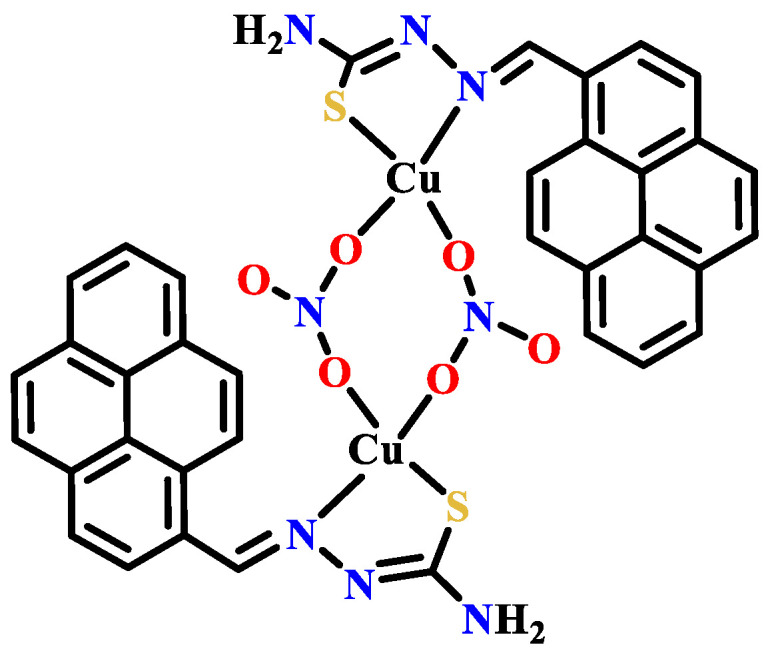
Binuclear copper(II) complex of pyrene hydrazone ligand with thiourea as a side chain bridged by two nitrate co-ligands.

Pyrene is another variety of important polycyclic aromatic hydrocarbons. However, due to the possible difficulties related to synthesis and the increased toxicity of pyrenehydrazone and its metal complexes, few related studies have been reported. In 2015, Suwarna A. Ingle et al. [133] reported a pyrene hydrazone ligand with thiourea as a side chain and its binuclear copper(II) complex (Figure 50). They fully characterized its structure by means of elemental analysis, ^1^H-NMR, IR, electrospray ionization—tandem mass spectrometry (ESI-MS), cyclic voltammetry, UV—Vis spectroscopy, and fluorescence spectroscopy. Similar to the anthrahydrazone mentioned above, the pyrenehydrazone ligand also coordinated with the central Cu(II) through the *N*/*S*-bidentate chelation mode, and the two nitrates acted as bridge ligands for the two Cu(II) through O atoms to form a symmetric, four-coordinated binuclear Cu(II) complex of pyrenehydrazone. The intercalation of pyrenehydrazone and the Cu(II) complex with DNA was expected due to the hyperconjugated planar structure of pyrene. In addition, the photo-induced cleavage (74%) of pBR-322 DNA by the Cu(II) complex was significantly stronger than that by the pyrenehydrazone ligand (14%). The study of the action mechanism showed that the complex could induce singlet oxygen during DNA cleavage. DFT calculations showed that the Cu(II) complex could generate singlet oxygen more efficiently than the ligand, and the highest occupied molecular orbital—lowest unoccupied molecular orbital (HOMO-LUMO) gap (0.332 eV) was smaller than that of the pyrenehydrazone ligand (0.629 eV). At the cellular level, the photo-toxicity of the complex and ligand to B16F10 melanoma cells was compared under the 365 nm of UV light and without UV light. The results showed that neither compound resulted in a large amount of cell death when incubated in the dark. However, cancer cells incubated with the Cu(II) complex had a significantly higher mortality rate than those incubated with the pyrenehydrazone ligand when exposed to 365 nm of UV light. The authors attributed this to the fact that the Cu(II) complex was more likely to produce singlet oxygen under photosensitization.

**Figure 51:**
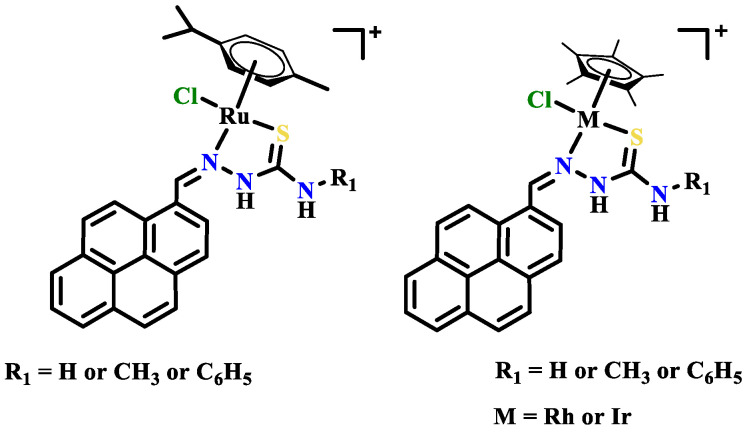
Organo-Ru(III)/Rh(III)/Ir(III) complexes of pyrene hydrazone with (*N*-methyl/phenyl)-substituted thiourea as the side chain.

Nandhagopal Raja et al. [134] also reported on a pyrene hydrazone ligand with thiourea as a side chain. The difference was that they introduced methyl and phenyl groups into the *N*-containing thiourea for SAR comparison. They further synthesized and characterized a series of new organometallic Ru/Rh/Ir complexes of pyrene hydrazone together using *p*-cymene or *η^5^*-C_5_Me_5_ as the co-ligand (Figure 51). The pyrene hydrazone ligand also chelated with the metal center via the *N*/*S*-bidentate mode. By comparing the results of the anticancer activity tests in vitro, it can be seen that the overall order of activity of the three pyrenehydrazone complexes was Rh(III) > Ir(III) > Ru(III), in which the Ru(III) and Ir(III) complexes showed weak inhibitory activity towards the tested cancer cell lines, with IC_50_ values ranging from 18 μM to 90 μM. The Rh(III) complex showed strong anticancer activity, with IC_50_ values in the range of 5~18.2 μM. For the same metal, the *N*-substituents of the thiourea side chain of pyrene hydrazone also had a weak effect on the activity of the complexes, and the order of activity was H > Ph > Me, which might also be related to the steric hindrance of the side chain. The highest activity was observed in the Rh(III) complex without the *N*-substituent, with IC_50_ values towards A549 and HeLa cells of 5.1 and 5.2 μM, respectively. Moreover, the toxicity of the Rh(III) complex towards HEK-293 human embryonic kidney cells was very low (166.5 μM), showing strong cytotoxicity selectivity. The Rh(III) complex with *N*-phenylthiourea as a side chain produced the second highest anticancer activity, with IC_50_ values of 9.3 μM for A549 cells and 9.6 μM for HeLa cells. Both of the Rh(III) complexes could arrest the A549 cell cycle in the G2/M phase and induced cell apoptosis. At 10 μM, both of the Rh(III) complexes induced 20% and 25% of apoptosis in A549 cancer cells.

**Figure 52:**
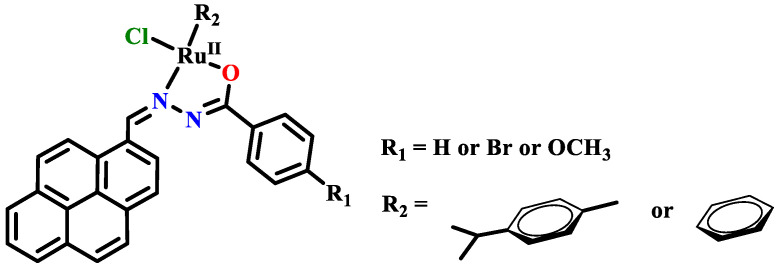
Organo-Ru(II) complexes of pyrene hydrazone with benzamide as a side chain together with benzene or *p*-cymene as a co-ligand.

Recently, Ramasamy Raj Kumar et al. [135] also reported two series of three half-sandwich ruthenium(II) complexes of pyrene hydrazone by using benzamide instead of thiourea as the side chain and either enzene or *p*-cymene as the co-ligand (Figure 52). The only structural difference of each series of complexes was that the *p*-substituents of benzoamide were H, Br, and -OCH_3_. In each complex, the pyrene hydrazone ligand coordinated with Ru(II) through the *N*/*O*-bidentate chelation mode. In addition to the aryl co-ligand, there was one Cl atom that acted as the potential leaving group in each complex, giving the whole complex a typical octahedral configuration. The structures of the complexes were characterized by IR, elemental analysis, and NMR. In vitro cytotoxicity screening was carried out for breast cancer cell line MCF-7 and lung adenocarcinoma cell line A549 together with the NIH-3T3 normal cell line. The results showed that the cytotoxicity of the six complexes towards NIH-3T3 cells was generally low. However, they showed different cytotoxicity levels to the two cancer cell lines. The IC_50_ values of the three Ru(II) complexes with a *p*-cymene co-ligand were in the range of 11.9~45 μM, stronger than those of the Ru(II) complexes with benzene as a co-ligand (IC_50_ = 30~50 μM). When the co-ligand was the same, the *p*-substituent group of benzamide affected the activity of the Ru(II) complexes in the order of H > OCH_3_ > Br. In particular, the Ru(II) complex with a *p*-cymene co-ligand and without a *p*-substituent had the most significant inhibition on the proliferation of A549, with an IC_50_ value of 11.9 ± 0.7 μM. It also showed much lower toxicity towards normal NIH-3T3 cells (IC_50_ = 225.3 μM), with a selectivity index (SI) of 18.9. These results indicated that the complex had significant cytotoxic selectivity against A549. In addition, the results of AO/EB and Hoechst 33258 cell staining and flow cytometry analysis showed that the Ru(II) complex caused cancer cell death mainly by inducing apoptosis. By analyzing the DNA content in the cell cycle distribution, it was found that the Ru(II) complex could block A549 cells in the G0/G1 phase.

In the past year or two, Carolina G. Oliveira et al. have reported similar work using *N*-substituted thiourea as a side chain and a pyrene hydrazone ligand of the same type that had been chelated with Pd(II) or Pt(II) to obtain corresponding metal complexes, in which ethyl and cyclohexyl are selected as side chain substituents.

**Figure 53:**
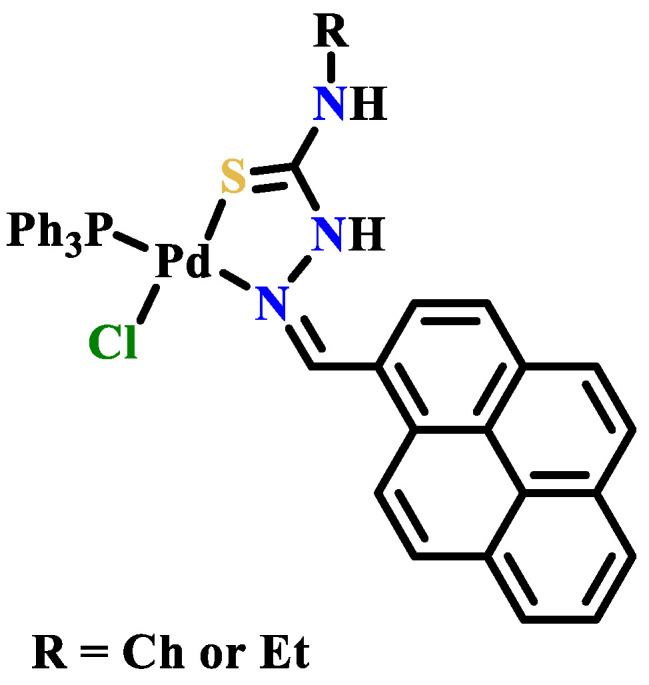
Pd(II) complexes of pyrene hydrazone with (*N*-ethyl/cyclohexyl)-substituted thiourea as a side chain.

In the past two or three years, Carolina G. Oliveira et al. [136,137] have also reported similar work as Nandhagopal Raja et al. [134], with *N*-substituted thiourea being used as the side chain of a pyrene hydrazone ligand and chelated with Pd(II) or Pt(II) to obtain corresponding metal complexes. Two four-coordinated Pd(II) complexes were first reported, with the main difference being that the substituents of the thiourea side chain are *N*-ethyl and *N*-cyclohexyl, respectively, for SAR. The other two coordination positions were occupied by PPh_3_ and Cl atoms, and their structures were characterized by mass spectrometry, NMR, elemental analysis, and other methods, as shown in Figure 53 [136]. Their antitumor activity and mechanism on human ovarian cancer line A2780 and the cisplatin-resistant strain A2780/cis were studied in vitro. The results showed that there was no significant difference in the anticancer activity of the two pyrene hydrazone ligands against the two cancer cell lines. After the coordination of pyrene hydrazone with Pd(II), the overall inhibitory activity of the compound was significantly increased, and the activity was closely related to the *N*-substituent group. The IC_50_ value of the *N*-ethyl-substituted Pd(II) complex towards the A2780 cell line was 0.74 μM, 10 times that of the *N*-cyclohexyl one (7.6 μM), showing a significant SAR. Interestingly, for the A2780/cis cell line, the IC_50_ of the *N*-ethyl-substituted Pd(II) complex was 35.8 μM, and its inhibitory activity was only 1/10 that of *N*-cyclohexyl (IC_50_ = 3.16 μM). In conclusion, the *N*-substituent of the thiourea side chain was very important for the anticancer activity and cell selectivity of the corresponding Pd(II) complex. Inductively coupled plasma mass spectrometry (ICP-MS) was used to detect the uptake of the Pd(II) complex in A2780 cells. The accumulation of the *N*-cyclohexyl-substituted Pd(II) complex was 7 ng, much higher than that of the *N*-ethyl-substituted one. At the same time, only 18% of the *N*-ethyl-substituted Pd(II) complex could cross the cell membrane, while the more lipophilic *N*-cyclohexyl one achieving 70% cell membrane penetration, although its cytotoxicity was still low, which might be also related to the steric hindrance of *N*-cyclohexyl. However, considering that the two complexes show opposite activity against A2780/cis cell line, it can be suggested that their anticancer mechanisms might be different. It would be interesting to further explore their action mechanisms at the molecular level.

**Figure 54:**
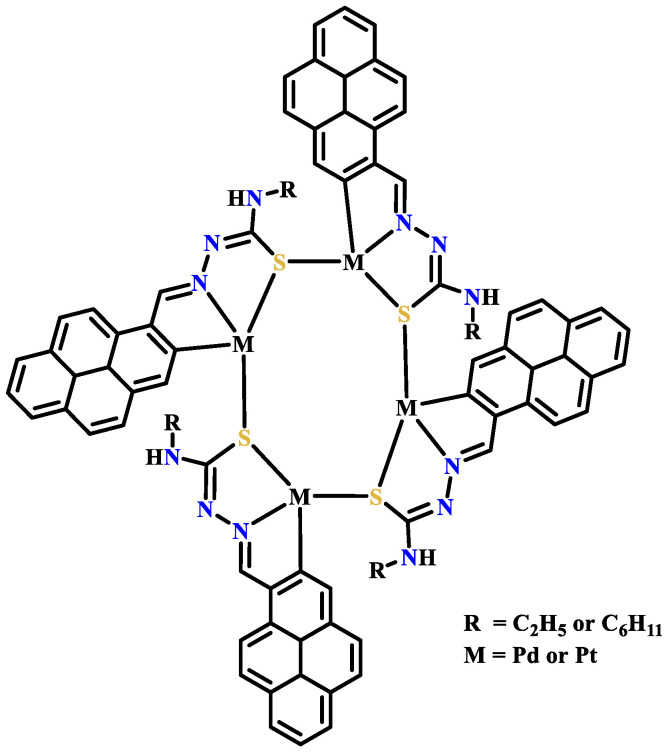
The novel symmetric tetranuclear Pd(II)/Pt(II) complexes of pyrene hydrazone with (*N*-ethyl/phenyl)-substituted thiourea as a side chain.

Subsequently, Carolina G. Oliveira et al. [137] further synthesized four symmetrical tetranuclear Pd(II) and Pt(II) complexes using the same pyrene hydrazone ligand, representing the first reported metal complexes of tetranuclear polycyclic hydrazones to date. The coordination structures of these complexes were consistent, as shown in Figure 54. Different from the above-mentioned mononuclear Pd(II) complexes of pyrene hydrazone, in these complexes, each pyrene hydrazone ligand not only coordinated with Pd(II)/Pt(II) through methylimide-N and thiol-S, but also coordinated with Pd(II)/Pt(II) through the 2′-C atom on the pyrene ring. Thus, the *C*/*N*/*S*-tridentate coordination pattern was formed, which is rare in the complexes of anthrachydrazone and pyrene hydrazone. In addition, each S atom acted as a bridging atom to connect two adjacent Pd(II)/Pt(II) to form a symmetric macrocyclic organometallic complex. The structure of each complex was characterized by IR, NMR, mass spectrometry (MS), and high-performance liquid chromatography (HPLC). The in vitro antiproliferative activities of pyrene hydrazones with a *N*-ethyl/*N*-cyclohexyl-substituted thiourea side chain and their four complexes on cervical cancer cell line A2780 and normal cell line MRC5 were compared based on SAR. The results showed that the Pd(II)/Pt(II) complexes with a *N*-ethyl substituent had higher activity, with IC_50_ values of 1.27 µM (Pd) and 0.37 µM (Pt), respectively, which were dozens of times higher than those substituted by *N*-cyclohexyl. They also had almost no toxicity to normal MRC5 cells (IC_50_ > 100 µM), showing excellent cytotoxicity selectivity, which was consistent with the activity trend of the mononuclear Pd(II) complexes above. The experimental results regarding the cell cycle, cell membrane integrity, and induction of cell apoptosis of these tetranuclear complexes shows that their anticancer mechanism was different from that of cisplatin, and they did not show significant effects on the anticancer target: DNA. Therefore, they might have good potential and prospects in overcoming drug resistance to cisplatin.

**Figure 55:**
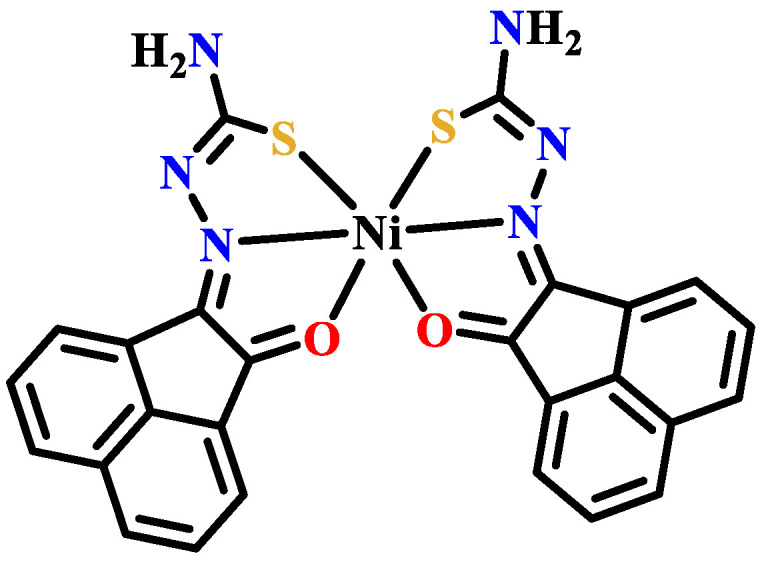
A six-coordinated nickel(II) complex by two same acenaphthehydrazone with thiourea as a side chain.

As early as 1997, Maria C. Rodriguez-Arguelles et al. [138] reported a new acenaphthehydrazone ligand with thiourea as a side chain and coordinated it with iron(II), nickel(II), copper(II), and zinc(II) to form four corresponding metal complexes (Figure 55). The structures were characterized by IR and NMR. It was observed for the first time that an acenaphthehydrazone ligand could inhibit cell proliferation and induce cell differentiation in the tested dose range. The acenaphthehydrazone ligand achieved 40% inhibition of DMSO-induced cell differentiation at 2 or 9.3 μg/mL, much higher than the corresponding ligands that had been previously reported, and failed to detect activity at higher doses (30 μg/mL). However, the Zn(II), Ni(II), and Fe(II) complexes showed a strong inhibitory effect on DMSO-induced cell differentiation, although they had no obvious inhibitory effect on cell proliferation at low doses. At low concentrations (2 μg/mL), they could achieve a 50~90% inhibition rate on DMSO-induced cell differentiation. Although the Cu(II) complex showed moderate activity, it was obviously lower than the metal complexes of other series of thiourea. Confusingly, the Cu(II) complex showed no inhibitory activity on DMSO-induced cell differentiation at the lower dose of 0.5 μg/mL, although the inhibition rate of the Cu(II) complex seemed to be the second highest among all of the compounds tested, as viewed from the histogram results. Additionally, the authors also did not test the activity of the Cu(II) complex at higher doses (such as 2 μg/mL). The results of the reverse transcriptase activity assay showed that the Zn(II) complex could promote the release of virions at 2 μg/mL, but the Cu(II) complex could not completely inhibit the retrovirus replication at the same concentration, with only 25% inhibition. However, the Ni(II) and Fe(II) complexes showed no activity during retrovirus release.

Overall, despite the research on the metal complexes of pyrene hydrazone and acenaphthehydrazone that has been carried out to some extent, when considering the limited studies regarding these anticancer metal complexes, the toxicity studies are still very insufficient, especially when considering that pyrene/acenaphthene is accepted as a type of carcinogenic compound. Thus, future research in this domain is necessary. Otherwise, the anticancer activity of pyrene hydrazone/acenaphthehydrazone and their metal complexes cannot be objectively recognized, as there is limited scientific evidence demonstrating their medicinal potential.

## 4. The Metal Complexes of Polycyclic Hydrazones for the Bioactive-Related Targets

In 2010, Yanling Xiang et al. [139] reported a 2-hydroxylnaphthylhydrazone ligand and its zinc(II) complex and studied the interaction between the Zn(II) complex and BSA using fluorescence and electronic absorption spectroscopy. In Tris-NaCl buffer solution with pH = 7.4, when the excitation wavelength was 370 nm, the fluorescence intensity of the Zn(II) complex was significantly enhanced at 475 nm after the addition of BSA. Conversely, the complex could quench the intrinsic fluorescence of BSA. According to the Stern—Volmer equation and the UV—Vis absorption spectra of the Zn(II) complex after adding BSA, the fluorescence-quenching mechanism was presumed to be static quenching.

**Figure 56:**
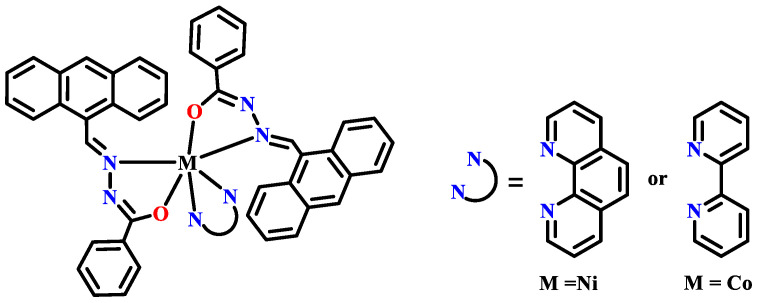
Ni(II)/Co(III) complexes of 9-anthrachydrazone with a benzamide side chain and with bipyridine or *o*-phenanthroline as a co-ligand.

In 2016, Satyajit Mondal et al. [140] reported on bipyridine with *o*-phenanthroline as a co-ligand and synthesized the Co(III) and Ni(II) complexes of 9-anthrachydrazone with benzamide as a side chain (Figure 56). Their structures were characterized by IR, UV—Vis, ESI-MS, and elemental analysis. The coordination mode was further determined by X-ray single-crystal diffraction analysis. The results showed that the coordination environment of the two metal centers was the same distorted octahedron. The two anthrahydrazone ligands coordinated with Co(III)/Ni(II) via *N*/*O*-bidentate mode as well as with bipyridine/*o*-phenanthrene via *N*/*N*-bidentate mode. This was also commonly found in the previous hydrazone complexes with bipyridine/*o*-phenanthroline as a co-ligand. UV—Vis absorption spectroscopy, fluorescence spectroscopy, and DNA viscosity experiments showed that the two complexes could bind with ct-DNA, but the binding mode was not the common intercalation mode, which was ascribed to the octahedral geometry of the complexes coordinated with the ligands in bidentate mode. In particular, the Co(III) complex with 3,5-di-tert-butylcatechol (3,5-DTBCH_2_) as a substrate showed significant catecholase mimicase activity. Kinetic determination showed that the oxidation rate of catechol followed the saturation kinetics relative to the substrate, and the k_cat_ value was very high: 1.00 × 10^5^ h^−1^. The authors carefully deduced the oxidation mechanism on the molecular level, and it was determined that the redox property of Co(III)/Co(II) plays a key role, and thus, the Ni(II) complex did not exhibit catecholase activity.

**Figure 57:**
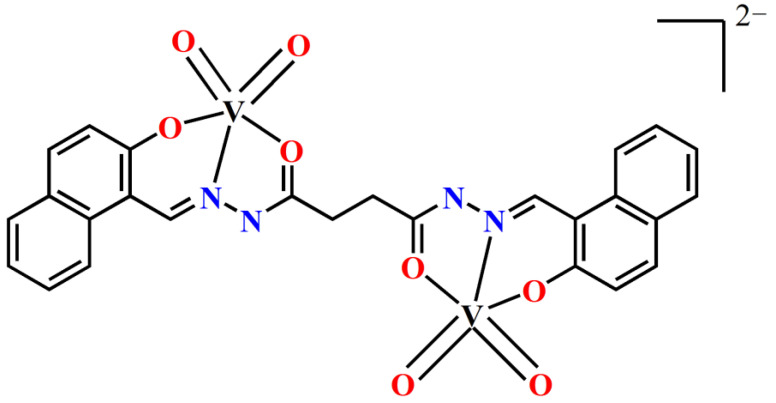
A symmetrical binuclear vanadium(V) complex of a special dinaphthylhydrazone ligand linked by the condensation of 1,4-succinylhydrazine with two 2-hydroxylnaphthaldehydes.

Subsequently, Sunshine Dominic Kurba et al. [141] also reported the biomimicase activity of a homobinuclear vanadium(V) complex involving 2-hydroxylnaphthylhydrazone (Figure 57). To be exact, it was actually a symmetrical dinaphthylhydrazone ligand joined by the condensation of 1,4-succinylhydrazine. The crystal structure and coordination mode of the complex were characterized and calculated by X-ray single-crystal diffraction analysis and density functional theory (DFT) analysis and were further compared with two similar vanadium(V) complexes of diphenylhydrazone. All three complexes were successfully used as model compounds of a functional catechin oxidase to oxidize 3,5-di-*tert*-butylcatechol to 3,5-di-*tert*-butyl-*o*-benzoquinone under the condition of an oxygen atmosphere. The system followed Michaelis–Menten kinetics with respect to the substrate. From the key kinetic parameters for the catalytic oxidation of the three complexes, the V_max_ of the naphthylhydrazone-V(V) complex was 1.47 × 10^−4^ M·s^−1^, which was lower than those of the other two phenylhydrazone-V(V) complexes (5.03 × 10^−4^ M·s^−1^ and 3.57 × 10^−4^ M·s^−1^). In contrast, the K_M_ value of the naphthylhydrazone-V(V) complex was 2.94 × 10^−4^ M·s^−1^, higher than those of the other two phenylhydrazone-V(V) complexes (1.60 × 10^−3^ M·s^−1^ and 1.42 × 10^−3^ M·s^−1^). In addition, the binding property of the V(V) complexes with BSA was studied by fluorescence spectroscopy. It was found that the binding affinity of the naphthylhydrazone-V(V) complex to BSA was 2.41 × 10^8^ M^−1^, which was also higher than those of the two phenylhydrazone-V(V) complexes (2.25 and 2.13 × 10^8^ M^−1^). 

**Figure 58:**
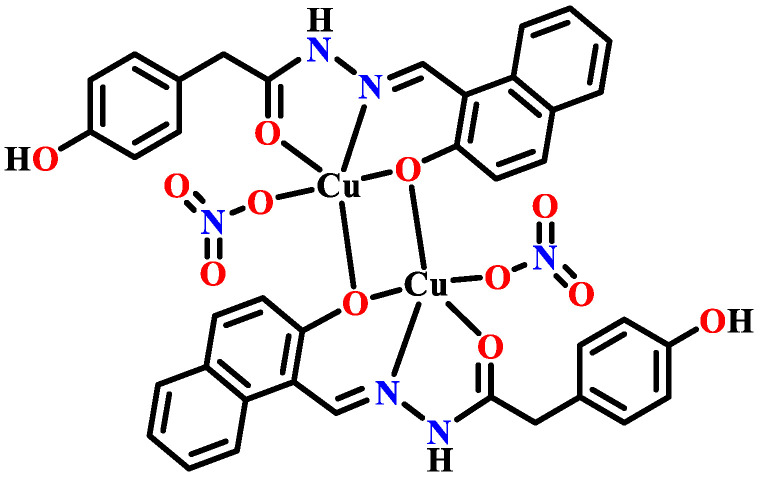
A symmetric binuclear copper(II) complex of 2-hydroxynaphthylhydrazone with *p*-hydroxyphenylacetamide as a side chain.

Meanwhile, Ming-Kun Yu et al. [142] reported a new 2-hydroxynaphthylhydrazone with *p*-hydroxyphenylacetamide as a side chain (Figure 58). A new binuclear copper(II) complex was synthesized by using it as the active ligand. The coordination environment of each Cu(II) can be regarded as a distorted rectangular pyramid, with each naphthylhydrazone coordinating with Cu(II) in an *O*/*N*/*O*-tridentate mode, in which the deprotonated hydroxyl-O atom acts as the bridging atom to link the two Cu(II) complexes. Another NO_3_^−^ on each Cu(II) occupies the top position of the pyramid. The coordination structure was determined by X-ray single-crystal diffraction analysis. The structural characteristics of the Cu(II) complex under different conditions were also studied by IR, UV—Vis, and electron paramagnetic resonance (EPR) spectral analyses. The magnetic properties of the complex were measured according to the EPR spectrum and magnetic susceptibility, which indicated that there was weak antiferromagnetic exchange and magnetic exchange between the two Cu(II) ions. The apparent activation energy of thermal decomposition showed that the Cu(II) complex had better thermal stability than the ligand. The results of the UV—Vis and DNA viscosity experiments showed that classical intercalative binding existed between ct-DNA and the naphthylhydrazone or its Cu(II) complex. The binding constant of the Cu(II) complex to ct-DNA was 6.24 × 10^6^ M^−1^, which was twice that of the ligand. The exothermic curves of their interactions with BSA were further measured by microcalorimetry. The results showed that both of them were endothermic reactions, and the reaction time was 27~42 min. The change in the interaction enthalpy (ΔH) of the Cu(II) complex bound with BSA was 30.3 kJ·mol^−1^, which was seven times that of the ligand. Subsequently, fluorescence spectroscopy was used to study the BSA binding properties. The results showed that the binding effect of the Cu(II) complex was more than twice that of naphthylhydrazone ligand, similar to the above DNA-binding effect.

**Figure 59:**
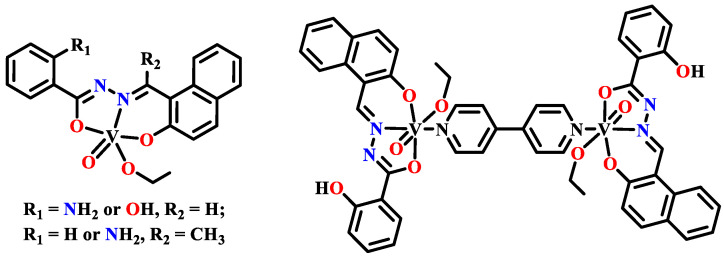
Some vanadium(V) complexes of naphthylhydrazone with an *o*-hydroxybenzamide side chain, reported by Rupam Dinda’s group.

As mentioned earlier, Rupam Dinda’s group has carried out valuable explorations in the study of the anticancer metal complexes of naphthylhydrazone. In addition, they have also reported four similar V(V) complexes of 2-hydroxylnaphthylhydrazone with *o*-hydroxybenzamide as a side chain and a further binuclear V(V) complex with 4,4′-bipyridine as a bridging ligand, as shown in Figure 59 below [143]. The structures and coordination modes of the complexes were characterized by various spectral analyses and X-ray single-crystal diffraction analysis, and the DNA/BSA-binding and catalytic properties of the complexes were studied. UV—Vis, cyclodextrin (CD), fluorescence spectroscopy, and thermal denaturation analysis showed that each complex could bind to the minor and major grooves of double-stranded DNA, and the binding constants were in the range of 10^4^~10^5^ M^−1^. The binding affinities of the two V(V) complexes when there was methyl substitution on the C atom (R_2_-) of the imine (C=N) of hydrazone were 7.16 × 10^5^ M^−1^ and 2.73 × 10^5^ M^−1^. The binding constants of BSA and BSA were in the range of 10^10^~10^11^ M^−1^. Moreover, the methyl-substituted complexes in the R_2_- position also had strong DNA photo-cleavage activity. The oxidative bromination of styrene and salicylaldehyde as well as the oxidation of methyl phenyl sulphide catalyzed by the five V(V) complexes were examined, all of which showed a high conversion ratio (>90%) and higher turnover frequency (TOF). In the oxidation bromination of styrene in particular, the conversion ratio and TOF reached the ranges of 96~98% and 8000~19,600 (h^−1^), respectively, indicating that these V^V^O complexes might be potentially good catalysts.

**Figure 60:**
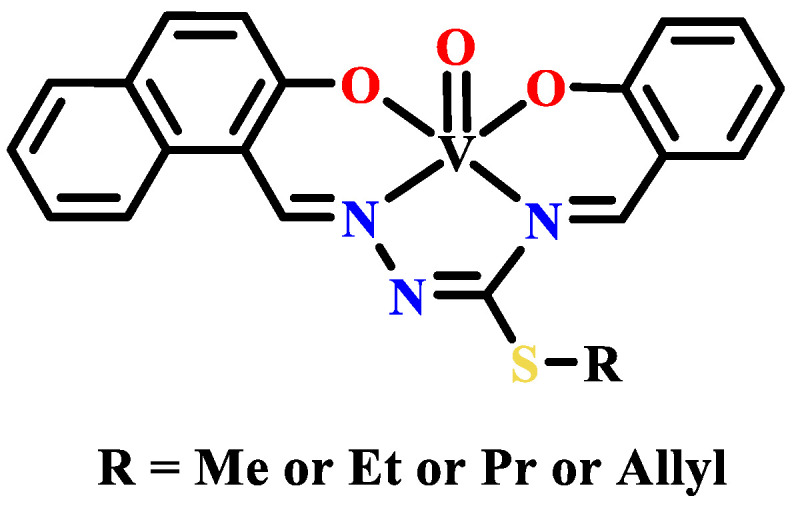
A series of oxovanadium(IV) complexes of naphthylhydrazone with different *N*-salicylic aldehyde thioureas as side chains.

Taking the antidiabetic oxovanadium complexes of N^1^,N^4^-diarylidene-thiosemicarbazidato’s ligand as a reference, Berat Ilhan-Ceylan et al. [144] designed a 2-hydroxylnaphthylhydrazone ligand with *N*-salicylic aldehyde thiourea as the side chain and synthesized four VO(IV) complexes of the same series of naphthylhydrazone by changing the *S*-substituents (methyl/ethyl/propyl/allyl) of thiourea (Figure 60). The structures and electronic states of the complexes were characterized by elemental analysis, IR, NMR and EPR. The EPR signals of the complexes both in the solid powder and solution state were detected and compared. The results indicated that all of them showed a single asymmetric line shape. A theoretical fitting study proved the presence of axial symmetry around the paramagnetic VO(IV). Cyclic voltammetry showed that there were two metal-based reversible redox peaks near 500 mV and −800 mV, corresponding to the V^IV^O/V^V^O and V^IV^O/V^III^O single-electron redox peaks, respectively. The range of 50~350 mV was considered to be the reduction reaction of the ligands. They also found that that the antioxidant capacity of the VO(IV) complexes increased with the increase in the carbon number in the saturated hydrocarbon on the *S*-substituent group of thiourea, so the VO(IV) complex with *S*-propyl had the highest antioxidant activity. These VO(IV) complexes were considered to be promising antidiabetic compounds.

**Figure 61:**
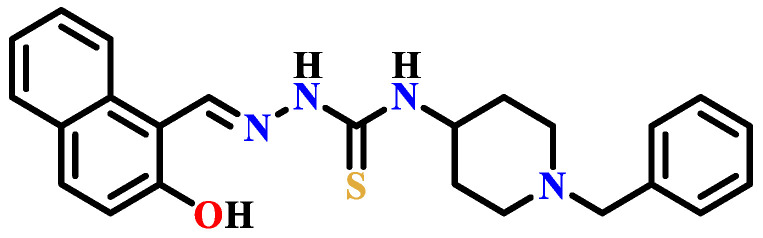
The chemical structure of NBPT containing naphthylhydrazone moiety.

Currently, there are more than 44 million people suffering from Alzheimer’s disease (AD) worldwide. However, there are currently only symptomatic treatments for AD, and there is no cure pathway. In view of the multifactorial pathogenesis of AD, there is still a lack of effective comprehensive treatment approaches, so it is necessary to develop new multi-target drugs. Recently, Duraippandi Palanimuthu et al. [145] reported a series of new *N*-benzylpiperidine thiosemicarbazide derivatives and their Cu(II)/Fe(III) complexes based on the pharmacophore (1-benzylpiperidine) of an acetylcholinesterase (AChE) inhibitor, donepezil, which also included a same-series derivative (NBPT) containing naphthylhydrazone moiety, as demonstrated in Figure 61 below. Of these compounds, pyridoxal 4-*N*-(1-benzylpiperidine-4-yl) thiosemicarbazoureas (PBPT) is the lead compound. These compounds were designed to address five key characteristics of AD, including low acetylcholine levels, dysfunctional autophagy, disrupted metal metabolism, protein aggregation, and oxidative stress. The authors primarily determined the in vitro anticancer activity tests for these compounds and then conduted systematic studies on anti-AD related mechanisms.

The proliferation inhibition activity towards SK-N-MC neuroepithelioma cells showed that NBPT showed moderate inhibitory activity among these compounds, with an IC_50_ value of 8.86 μM; however, that of the Fe(III) complex of NBPT was only 52.90 μM, while that of the Cu(II) complex reached 1.81 μM. This should be related to the cytotoxicity of Cu(II) itself. Further studies on the anti-AD mechanism did not involve the metal complexes. Alone, NBPT showed differences in the examined anti-AD mechanisms. For example, it had a relatively significant iron-chelating efficiency, inhibited copper-mediated amyloid B aggregation, failed to inhibit H_2_O_2_-mediated cytotoxicity due to its own cytotoxicity, and, together with moderate AChE inhibitory activity, it induced autophagy. These properties provide scientific data for the lead compound, PBPT, as a promising and potential multifunctional anti-AD agent.

**Figure 62:**
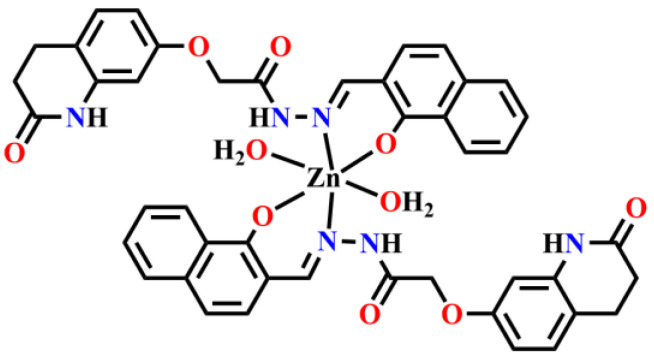
A symmetric Zn(II) complex of two of the same 1-hydroxylnaphthylhydrazones with an acylhydrazone derivative as a side chain.

Mustapha C. Mandewale et al. [146] also designed and synthesized a series of acylhydrazone derivatives based on the key anti-tuberculosis pharmacophore, 3,4-dihydroquinolin-2(1H)-one, as well as Zn(II) complexes, in which a derivative of 1-hydroxynaphthylhydrazone and its corresponding Zn(II) complex was included (Figure 62). Their structures were characterized by IR, NMR, and elemental analysis. Regarding the Zn(II) complex of naphthylhydrazone, two naphthylhydrazone ligands coordinated with the central Zn(II) through the *N*/*O*-bidentate chelation mode via azomethine-N and phenyl-O, and two H_2_O were also involved to form a typical octahedral coordination configuration. The Alamar Blue method was used to detect the primary anti-tuberculosis activity of the compounds. The results showed that these Zn(II) complexes showed significant inhibitory activity against *Mycobacterium tuberculosis*. The inhibition rate of the Zn(II) complex of this only naphthylhydrazone was 84.1% at 3.12 μg/mL, while the inhibition rate of the ligand was only 2.6%. When the concentration increased to 12.5 μg/mL, the Zn(II) complex could reach 99.2%. However, the anti-tuberculosis activity of this Zn(II) complex of naphthylhydrazone was not the best according to a comprehensive comparison.

**Figure 63:**
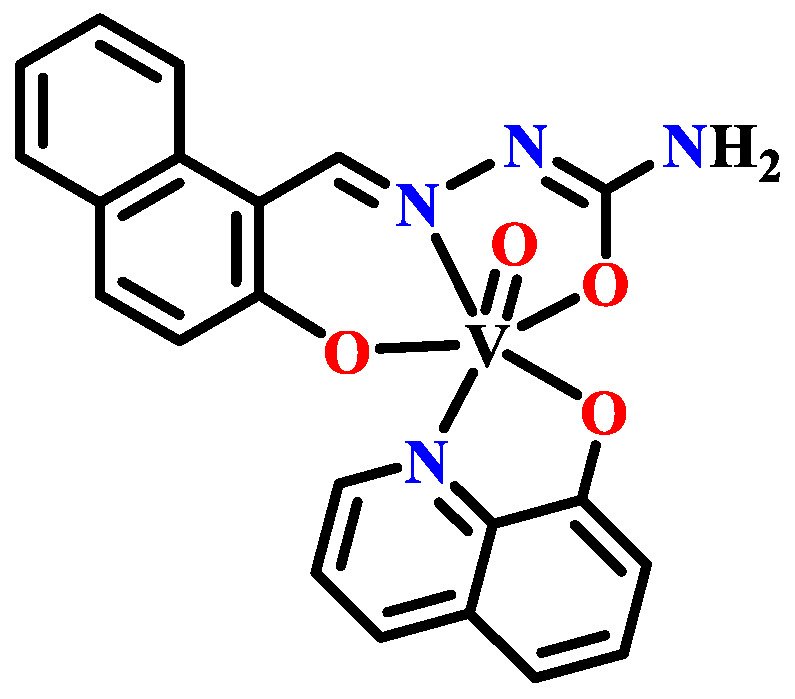
A new oxovanadium(V) complex of naphthylhydrazone with a urea side chain and 8-hydroxyquinoline (8-HQ) as a co-ligand.

At the same time, Gonzalo Scalese et al. [147] reported a new mixed-ligand oxovanadium(V) complex using 2-hydroxynaphthylhydrazone with a urea side chain and 8-hydroxyquinoline (8-HQ) as two bioactive ligands in order to find new vanadium(V) complexes against *Trypanosoma cruzi*. An SAR study was also performed by comparing the V(V) complexes of three similar 2-hydroxyphenylhydrazone ligands (Figure 63). The structures of these V(V) complexes were characterized in both the solid and solution states. In each complex, the central VO(V) was chelated by the *O*/*N*/*O*-tridentate hydrazone ligand and *N*/*O*-bidentate 8-HQ. The authors further investigated the bioactivity of *Trypanosoma cruzi* superembryos (CL Brener) and Vero cells as mammalian cell models. The results showed that the IC_50_ of these mixed-ligand VO(V) complexes against *T. cruzi* ranged from 6.2 to 10.5 μM, which was similar to the activity of nifurtimox and 8-HQ, but 4~7 times higher than that of the free hydrazone ligand. Comparatively, the VO(V) complex ([V^V^O_2_(L-H))]) with only the hydrazone ligand had little inhibitory activity and low selectivity against parasites. Thus, the introduction of the 8-HQ co-ligand significantly improved the activity and selectivity. The parasites treated with the complex did not show a late apoptotic or necrotic phenotype, suggesting a different mechanism of cell death. In vivo toxicity studies on zebrafish models showed that the most representative VO(V) complex with the highest antiparasitic activity, although not with the naphthylhydrazone ligand, showed non-toxicity at higher concentrations of 25 µM. These results indicate that this variety of VO(V) complexes has good medicinal prospects for the treatment of parasitic diseases.

## 5. Conclusions

**Scheme 1:**
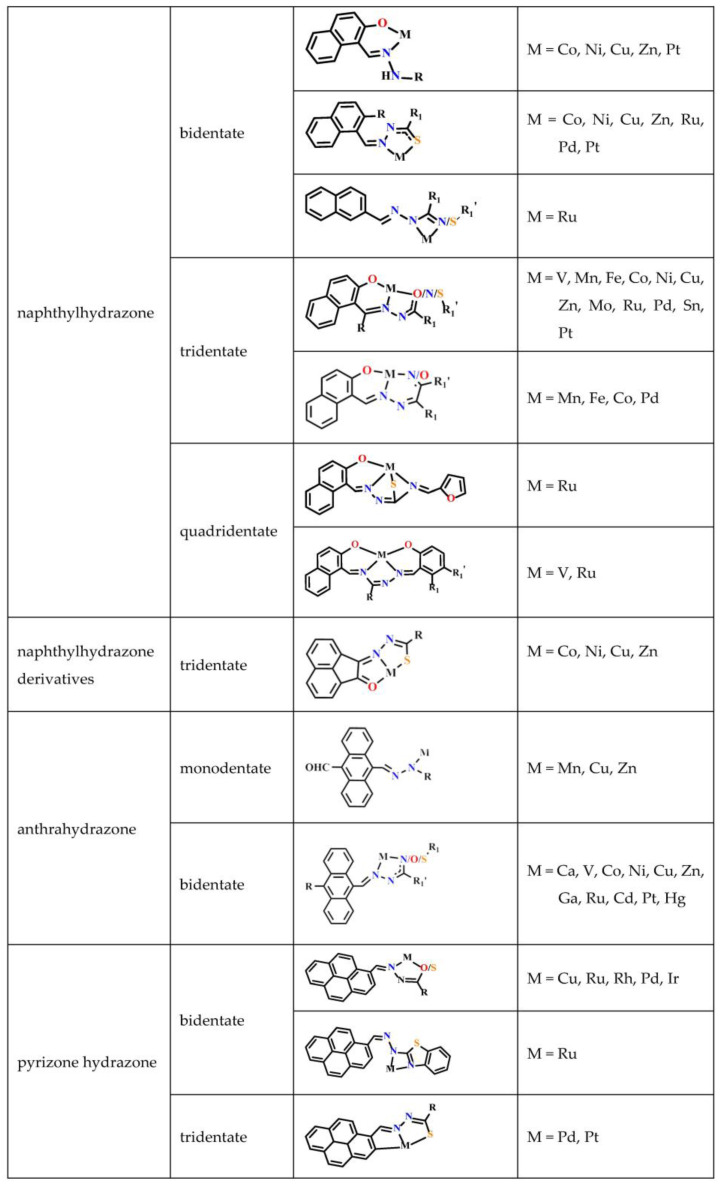
An overview scheme showing various coordination modes of most of the polycyclic aromatic hydrazone metal complexes mentioned within.

According to the overall research results regarding the metal complexes of polycyclic aromatic hydrazones, we have created a preliminary understanding on their explicit biological activities and potential medicinal prospects. Considering that there are abundant ways to design and synthesize metal complexes, there is still broad space for further exploration and discovery. From the results reported here, we have also realized some characteristics and shortcomings, especially regarding SAR, including the following:

1. We found that there is a significant structure—activity relationship for the anticancer activity of the metal complexes of hydrazones, but this relationship is very complicated because the influencing factors include the central metal, number of hydrazone rings, side chain groups, substituents of the side chain groups, and co-ligands (as illustrated in Scheme 1). Therefore, in order to further understand and clarify the structure—activity relationship of these anticancer metal complexes, it is necessary to guide the rational design of metal drugs and to synthesize and test more aromatic hydrazone metal complexes with anticancer activity. However, through the combination of available reports presented here, we have found some rules and characteristics regarding SAR on the metal complexes of polycyclic aromatic hydrazones. From the perspective of side chains, thiourea and acylhydrazone showed better bioactivity, which seems to be related to the addition of auxiliary chelating sites. Furthermore, this also makes the complexes more stable, which contributes to exerting the synergistic effect between the central metal and the hydrazone. In addition, a more alkaline *N*-heterocyclic (such as imidazoline) seems to be beneficial to increase the activity if the side chain has a *N*-heterocyclic pharmacophore, as suggested by our reported and unreported results [123]. Considering that it is easier for a stronger alkaline N atom to be protonated into quaternary ammonium salt, it is speculated that this could be related to the influence of the charged property of the side chain when acting on its potential intracellular targets. In addition, when viewed from the metal center, the copper(II) complexes of such hydrazones exhibited significantly higher pharmacological activity, which was primarily ascribed to the bioactivity of copper(II) itself. However, the potential toxicity of copper(II) complexes should also be considered seriously. Other metal centers, however, did not show distinct and predictable activity patterns, which means that different metal complexes of polycyclic aromatic hydrazones are still worth exploring more widely.
2. Furthermore, according to the complexes reported in this review, the antitumor and antibacterial activities of the complexes of naphthylhydrazone were almost found to be higher than those of the corresponding phenylhydrazone [58,76,78,85], although the metal complexes of phenylhydrazone were not specifically discussed here. In addition, when used as co-ligands, *o*-phenanthroline and bipyridine could effectively improve the antitumor activity, and *o*-phenanthroline was even better than bipyridine, as reported in the mentioned vanadium and ruthenium complexes as well as in some instructional reviews [49,50,76,78,85,129]. In fact, this pattern was also observed in our unreported study on the metal complexes of anthrahydrazone. We think that the expansion of the aromatic ring and the addition of the co-ligand obviously increase the lipophilicity of the complex, which could enhance its ability to penetrate the cell membrane and thus improve its absorption efficiency by cells or bacteria. However, the metal complexes of anthrahydrazone should not be taken lightly. Although it is generally believed that the toxicity and carcinogenicity risks of anthracyclines increase as the number of cycles increases, it is worthwhile to further enrich the metal complexes of anthrahydrazone by considering the successful application of anthracyclines such as doxorubicin. We also think that aromatic hydrazone compounds with more than four rings are not only rarely studied, but also do not exhibit the prospect of medicinal applications due to their obvious high toxicity and poor solubility.
3. When testing the anticancer activity, more normal human cells should be selected as cell lines to reflect the cytotoxicity of the metal complexes of naphthylhydrazone more comprehensively. Furthermore, it is regrettable to note that many of the metal complexes of naphthylhydrazone that have been reported to show very high anticancer activity in vitro have not been thoroughly studied and evaluated for their anticancer activity in animals. It is suggested that further work should be conducted in this area to promote research on the drug properties of hydrazone metal complexes, especially those candidate complexes that have shown high activity in vitro.

In this review, for the first time, we summarized the studies on the biological activity, such as the antibacterial and anticancer activity, of various metal complexes of polycyclic aromatic hydrazones in combination with our own work on the anticancer metal complexes of anthrahydrazone. It should be mentioned that the work in this direction has not received much attention. On the other hand, there is much more research on the biological activity of the metal complexes of phenylhydrazone than those of polycyclic aromatic hydrazones. Due to energy limitations, we were unable to provide a corresponding review and introduction. It is hoped that experts who are interested in this topic will be able to carry out further work and provide higher-level direction and guidance for researchers in this field.

## Data Availability

Not applicable.
